# Activation of Ethanol Transformation on Copper-Containing SBA-15 and MnSBA-15 Catalysts by the Presence of Oxygen in the Reaction Mixture

**DOI:** 10.3390/ijms24032252

**Published:** 2023-01-23

**Authors:** Izabela Sobczak, Joanna Wisniewska, Piotr Decyk, Maciej Trejda, Maria Ziolek

**Affiliations:** Faculty of Chemistry, Adam Mickiewicz University in Poznan, Uniwersytetu Poznanskiego 8, 61-614 Poznan, Poland

**Keywords:** copper and copper-manganese SBA-15, FTIR study in flow system, ethanol dehydrogenation to acetaldehyde, the role of oxygen in the enhancement of activity, reaction pathways

## Abstract

The aim of this study was to get insight into the pathway of the acetaldehyde formation from ethanol (the rate-limiting step in the production of 1,3-butadiene) on Cu-SBA-15 and Cu-MnSBA-15 mesoporous molecular sieves. Physicochemical properties of the catalysts were investigated by XRD, N_2_ ads/des, Uv-vis, XPS, EPR, pyridine adsorption combined with FTIR, 2-propanol decomposition and 2,5-hexanedione cyclization and dehydration test reactions. Ethanol dehydrogenation to acetaldehyde (without and with oxygen) was studied in a flow system using the FTIR technique. In particular, the effect of Lewis acid and basic (Lewis and BrØnsted) sites, and the oxygen presence in the gas reaction mixture with ethanol on the activity and selectivity of copper catalysts, was assessed and discussed. Two different reaction pathways have been proposed depending on the reaction temperature and the presence or absence of oxygen in the flow of the reagents (via ethoxy intermediate way at 593 K, in ethanol flow, or ethoxide intermediate way at 473 K in the presence of ethanol and oxygen in the reaction mixture).

## 1. Introduction

Ethanol transformation into different valuable products is of great interest at present because bioethanol is obtained from renewable resources (biochemical conversion of lignocellulosic feedstocks to ethanol) and, therefore, its utilization is not expensive. Acetaldehyde is obtained by dehydrogenation of ethanol and acetaldehyde is an intermediate that can be used for the formation of other compounds, e.g., 1-butanol, ethyl acetate, or 1,3-butadiene whose syntheses comprise a few steps and require catalysts containing acidic and basic active centers. From among them, 1,3-butadiene is an important compound as, thanks to the conjugated double bonds, these highly reactive molecules are involved in numerous chemical processes (e.g., in Diels–Alder, dimerization and oligomerization, hydrogenation and oxidation reactions, polymerization to synthetic elastomers-styrene butadiene rubber, and polybutadiene rubber) [1]. One of the ways for further transformation of butadiene is the oxidation to maleic anhydride, applied in the production of unsaturated polyester resins used in thermosetting plastics, such as reinforced fiberglass for construction products, and Kevlar for protective wear. It is also an important feedstock in the manufacture of copolymers and lubricating oil additives [2]. The catalytic conversion of ethanol to 1,3-butadiene is a multistep process. It involves several consecutive stages: (1) dehydrogenation; (2) aldol condensation; (3) crotonic condensation; (4) Meerwein–Ponndorf–Verley (MPV) reduction; and (5) dehydration [1]. The first step of this reaction is the dehydrogenation of alcohol to form acetaldehyde. The reaction occurs typically on basic centers and often is a reaction rate-limiting step. The competitive reaction is the dehydration of alcohol to dimethyl ether and ethene, which proceeds on strong acidic sites. Therefore, catalysts with weak acidity and strong basicity are welcome for the achievement of high selectivity in the production of 1,3-butadiene.

It has been proposed by several authors [1,3,4,5] that on metal oxides, ethanol dehydrogenation to acetaldehyde (the first step in 1,3-butadiene production) proceeds via surface ethoxy (neutral CH_3_CH_2_O chemisorbed species) [6] or ethoxide (deprotonated ethanol anion [CH_3_CH_2_O]^−^) [3,4,5] intermediates. Ethoxy species are also intermediates in the formation of ethene via dehydration reaction [7] and, therefore, the composition of the active sites on the catalysts surface dedicated for acetaldehyde production from ethanol should be chosen so that it would block the undesired reaction path towards ethene formation (elimination of strong acid sites). For the reactions over metal oxides and metallic copper catalysts [6,8], a sequential mechanism involving the dissociation of ethanol adsorbed on an acid-strong base pair site to ethoxy species, and subsequent elimination of a proton (α-hydrogen) in the ethoxy group to form acetaldehyde has been proposed.

However, as it was summarized in a review paper [1], the formation of acetaldehyde can proceed also via a different way in which the surface hydroxyls of the metal support can take part in the acetaldehyde formation. On silica-supported silver, the reaction has been proposed to proceed via the simultaneous proton abstraction of an H-bonded complex formed between ethanol and surface silanol groups [1,9]. Pomalaza et al., in their review [1], have underlined that further studies should be performed to confirm the nature of surface intermediates on the various catalysts dedicated for the Lebedev process (one-step ethanol to butadiene reaction) which preferably requires a bifunctional acid-base catalyst. Their suggestion has been a starting point for our study.

The focus of our study was on unraveling the pathway of acetaldehyde formation from ethanol (the rate-limiting step in 1,3-butadiene production) on copper catalysts supported on mesoporous silica, i.e., the catalytic system containing metal oxide and a high concentration of hydroxyls on the surface of the support. For this purpose, we used ordered mesoporous silica SBA-15 and SBA-15 doped with manganese as supports for copper, and the focus was on a study of their activity and selectivity in ethanol dehydrogenation to acetaldehyde (the first step in 1,3-butadiene preparation from ethanol), also in the presence of oxygen. The choice of SBA-15 as a support for copper species was dictated by a large number of silanol groups on the surface and a large surface area available for the dispersion of copper oxide active species. Such a support would permit estimation of the role of surface hydroxyls in the ethanol to acetaldehyde transformation, underlined in [9] for silver/silica catalyst. Manganese was used as a dopant for SBA-15 support in order to create copper-manganese synergistic interaction allowing modification of the strength of acid-base pair in copper oxide active species (a decrease in Lewis acidic sites strength-copper cations, and an increase in the strength of basic sites-oxygen ions). The important part of our work was to provide a detailed insight into the effect of oxygen present in a gas mixture, with ethanol on the activity and selectivity of copper catalysts based on SBA-15 and MnSBA-15 supports in the reaction of ethanol to acetaldehyde dehydrogenation. We wanted to answer the question: (i) if oxygen changes the active centers on the catalyst surface by oxidation of metal species, (ii) modifies their activity by adsorption on the catalyst surface to form additional base sites, or (iii) takes part in the electron transfer between copper cations with different oxidation states.

## 2. Results and Discussion

### 2.1. Composition and Structure/Texture of Catalysts

Table 1 shows the contents of metals (Mn, Cu) in the catalysts prepared as measured by the ICP-OES method. The results clearly show that the actual loading of manganese in the SBA-15 sample was much lower than expected (Section 3.2)-0.5 wt.%. It indicates the difficulty of Mn introduction in higher amounts into the SBA-15 structure during the hydrothermal synthesis (despite lowering the acidity (pH = 3) of the synthesis mixture). On the other hand, copper loading in mesoporous silica was as expected (2.0–2.2 wt.%), which confirmed that the grafting of silica with organosilane is an effective method for metal introduction. However, this two-step modification (grafting with (3 aminopropyl) trimethoxysilane (APTMS) in toluene and copper anchoring) led to a slight decrease in Mn loading in MnSBA-15 support.

XRD and N_2_ ads./des. studies were performed in order to characterize the structural/textural properties of SBA-15 catalysts. In the XRD patterns of SBA-15 and MnSBA-15 materials, recordedh before and after modification with copper (Supplementary Figure S1), three typical and well-resolved diffraction peaks appeared at 2 theta ca. 1°, 1.6°, and 1.8° assigned to Miller indexes (100), (110) and (200) [11] confirming that the samples obtained had a hexagonally ordered structure typical of SBA-15 mesoporous material. The (100) index is characteristic of hexagonally ordered pores in an SBA-15 structure, while the (110) and (200) indices are typical of the pores well-ordered in long-range [12]. It is worth noting that the peak at ca. 1° in the Cu-SBA-15 pattern was shifted to the higher value of 2 theta in comparison to that in the SBA-15 pattern, suggesting a decrease in the pores size as a result of copper modification of the support. In the wide angle range of the XRD patterns of Cu-SBA-15 and Cu-MnSBA-15, no reflections from copper crystallites appeared, indicating high dispersion of copper on the silica surface.

The mesoporous character of the samples prepared was confirmed by the N_2_ adsorption/desorption isotherms (Supplementary Figure S2), which were of type IV(a) of IUPACs classification [13], typical of mesoporous materials. SBA-15 and MnSBA-15 samples showed large specific surface areas (870–940 m^2^/g). The averaged pore volume and pore diameter of both supports were similar (ca. 1 cm^3^ g^−1^ and 11 nm, respectively). However, a significant decrease in the surface area and pore volume was observed for the samples modified with copper (surface area ca. 420–660 m^2^/g, pore volume ca. 0.6 cm^3^/g). The decrease in the surface area and pore volume can be a result of the functionalization of mesoporous solids with APTMS before modification with metal [14,15] and/or copper location in the pores of SBA-15. As concerns the pore diameter, a higher decrease in this parameter was observed for Cu-SBA-15. It is in agreement with the XRD results.

### 2.2. State of Metals

The state of metals (copper and manganese) in the catalysts was determined on the basis of ultraviolet-visible (Uv-vis) spectroscopy, X-ray photoelectron spectroscopy (XPS), and electron paramagnetic resonance (EPR) measurements.

**Figure 1:**
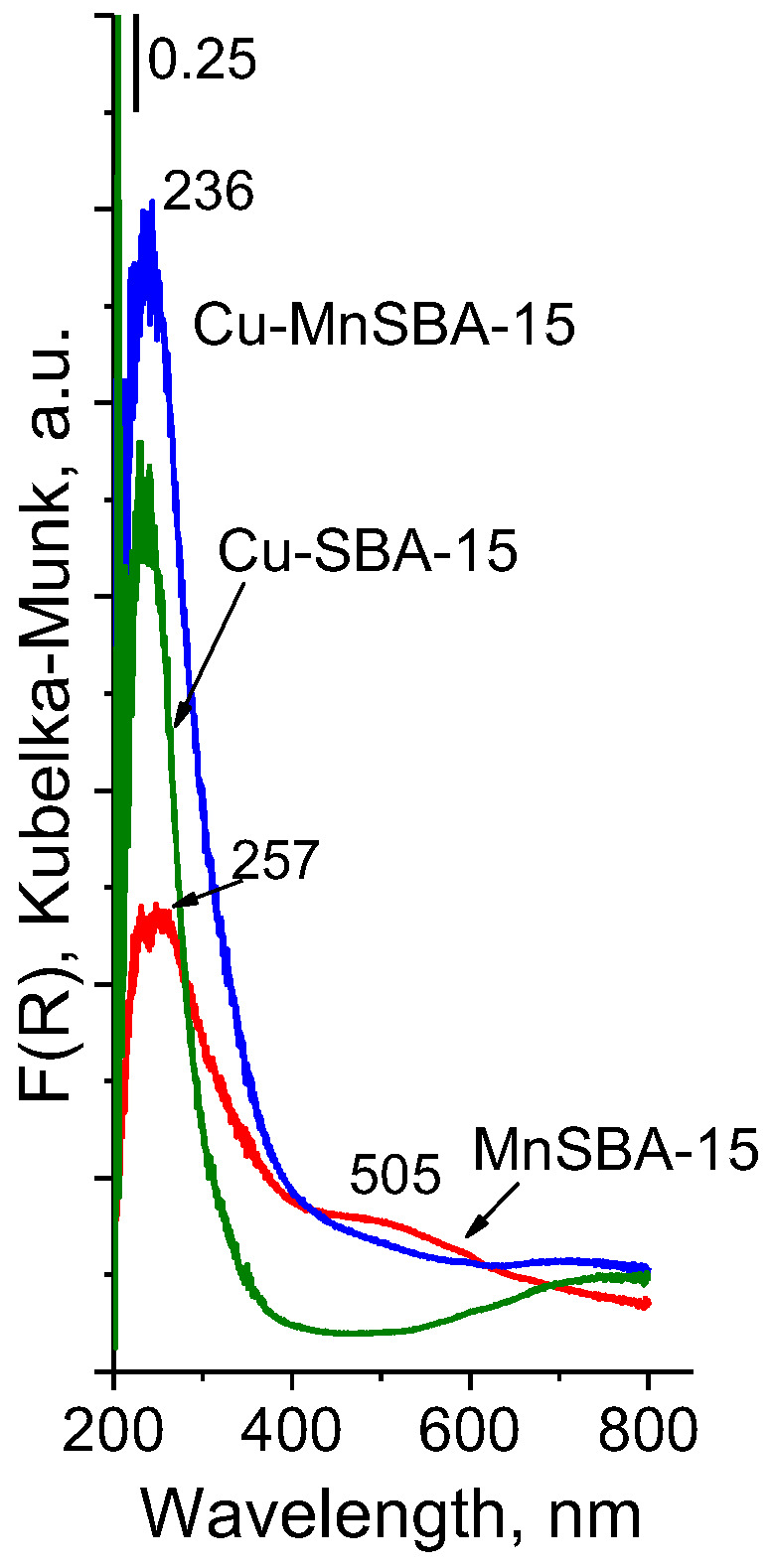
Uv-vis spectra of MnSBA-15 and copper-containing SBA-15 and MnSBA-15 materials.

A comparison of Uv-vis spectra of MnSBA-15 and Cu-MnSBA-15 is presented in Figure 1. The band at ca. 500 nm observed in the MnSBA-15 sample is assigned to the d-d transition of Mn^3+^ in octahedral coordination [16,17]. The band at ca. 250 nm contains two maxima at ca. 230 and 260 nm to charge transfer transition between O^2−^ and Mn^2+^/Mn^3+^ [10,16,17,18] in tetrahedral (isolated, located in the skeleton of silica) and octahedral coordination (extra framework) manganese species. In Cu-MnSBA-15, the broad band at ca. 250 nm is overlapped with the intense band at ca. 230 nm originating from cationic copper species (Cu^2+^). The second broad band at ca. 700–800 nm observed in the spectrum of Cu-MnSBA-15 can be assigned to copper oxide (CuO) species [19]. Interestingly, the band at ca. 500 nm, characteristic of MnSBA-15, is considerably diminished in the spectrum of Cu-MnSBA-15, suggesting the partial removal of extra framework manganese species during modification of the sample with copper source or their covering with copper species. The latter suggestion seems to be more probable because the decrease in Mn content after copper loading is very low (0.02 wt.%-Table 1) and can be caused by the introduction of an additional component (copper–2.2 wt.%). Thus, one can postulate that copper and manganese extra framework species are in the neighborhood and can interact synergistically.

The spectrum of Cu-SBA-15 material shows a less intensive (because of the lack of the band coming from manganese species) band at 230 nm (from isolated cationic copper species (Cu^2+^)), and a band at ca. 750 nm characteristic of copper oxide (CuO) species. A slightly visible shift of the latter band to a lower wavelength in the spectrum of Cu-MnSBA-15 suggests the interaction between CuO and manganese species.

**Figure 2:**
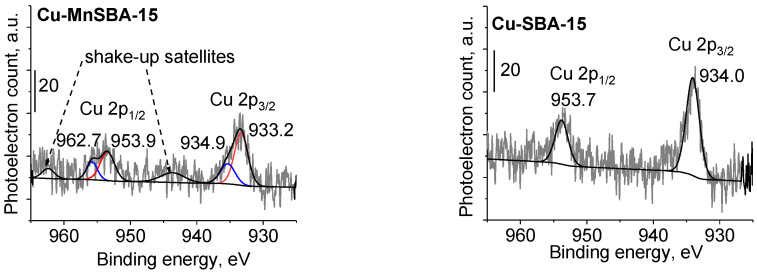

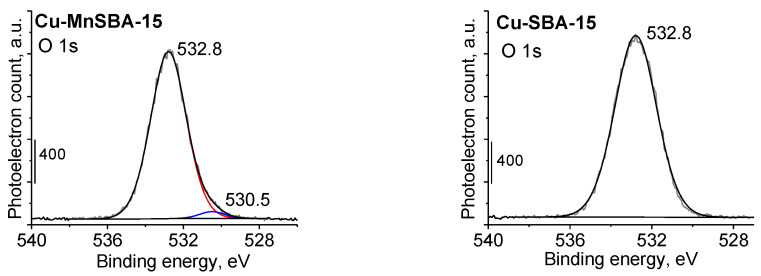
Cu 2p and O 1s regions of XP spectra of copper-containing SBA-15 and MnSBA-15 catalysts.

In order to get more insights into the surface composition and electronic state of the catalyst components, X-ray photoelectron spectroscopy was applied. All XP spectra were normalized to the binding energy (BE) of Si 2p = 103.4 eV. The Cu 2p_3/2_ and Cu 2p_1/2_ core level were employed to investigate the Cu surface oxidation and the XP spectra of Cu-SBA-15 and Cu-MnSBA-15 are shown in Figure 2.

As reported in the literature [20,21], XPS peaks at 931–933 eV and 933.5–935.5 eV are typical of Cu 2p_3/2_ core level of Cu^+^ and Cu^2+^ species, respectively. The binding energy gap between Cu 2p_3/2_ and Cu 2p_1/2_ core level should be ca. 20 eV. It is necessary to note that it is difficult to distinguish the Cu^+^ ion from zero-valence copper by XPS, but Cu^2+^ species can be easily identified by the presence of the satellite signals attributed to an electron transfer from a ligand orbital to the d orbital of the metal [22].

As shown in Figure 2, the binding energies in the Cu 2p_3/2_ and Cu 2p_1/2_ regions for Cu-SBA-15 are 934.0 eV and 953.7 eV, respectively. The position of the bands suggests the presence of Cu^2+^ species, however, the absence of the satellite peaks at higher binding energies of ca. 9 eV excludes the presence of copper(II) oxide. It is possible that the valence state of Cu is between +1 and +2, as already mentioned in the literature [23]. The deconvolution of the XP spectrum of Cu-MnSBA-15 reveals two bands in the Cu 2p_3/2_ region: at 933.2 eV and 934.9 eV. They are correlated with the bands in the Cu 2p_1/2_ region (953.9 eV and 962.7 eV, respectively). The first pair of bands (933.2 eV and 953.9 eV) indicates the presence of copper species at a lower oxidation state. The binding energies are higher than reported in the literature for Cu_2_O [20,21].

The second pair of XP bands (934.9 eV and 953.9 eV) is correlated with the slight shake-up satellite peaks (ca. 944 eV and ca. 962 eV) and evidence of the presence of bivalent copper (Cu^2+^), probably in copper oxide(II). The presence of copper oxide in the Cu-MnSBA-15 sample was also confirmed by the oxygen XP spectrum (Figure 2). This spectrum is asymmetrical, and therefore, it was deconvoluted into two bands at 532.8 eV and 530.5 eV. The first band is more intense and comes from the oxygen in the silica structure (Si-O-Si bond) [24]. The second one can be assigned to the oxygen in copper oxide [25,26]. The oxygen (O 1s) XP spectrum of Cu-SBA-15 displays only one band located at ca. 532.8 eV, which is assigned to the oxygen in the Si-O-Si bonds from the silica structure.

Thus, XPS results showed copper in Cu-SBA-15 to be present as Cu^δ+^ (1 < δ < 2) and/or isolated Cu^2+^ whereas in Cu-MnSBA-15 copper is present in both oxidation states Cu^δ+^ (1 < δ < 2) and Cu^2+^ in copper oxide(II). The change in BE of copper species in the presence of manganese in the Cu-MnSBA-15 catalyst suggests some interaction between copper and manganese species. It has to be pointed out that XPS analyses are performed after the evacuation of the samples at room temperature.

On the basis of the presented XP spectra and XP spectra of the other catalyst components (Si, O, C), the external surface concentration of copper has been calculated. For Cu-SBA-15, the surface concentration of copper is 1.19 wt.%, whereas, for Cu-MnSBA-15 this value is equal to 0.79 wt.%.

**Figure 3:**
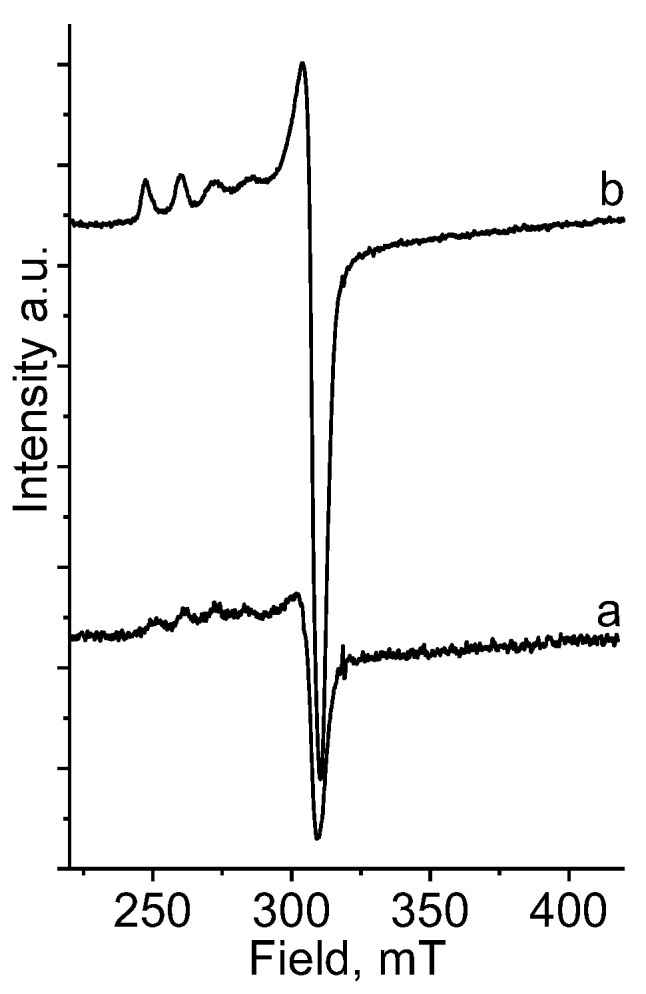
EPR spectra of Cu-SBA-15 recorded at 293 K (a) and 77 K (b) for the fresh sample.

Moreover, EPR studies were performed to identify the copper species in fresh Cu-SBA-15 and the sample after evacuation at 623 K. The spectra recorded at 293 K and 77 K are shown in Figure 3.

The visibly axially symmetric EPR spectra originated from isolated Cu^2+^ cations in octahedral coordination. The spectrum recorded at 77 K corresponds to the spin–Hamiltonian parameters, i.e., the values of the tensor g_ǁ_ = 2.385, hyperfine structure (hfs) lines A_ǁ_ = 130.5 G, characteristic of copper in an environment of a symmetry close to that of perturbed octahedron [27]. Cu(II) complexes supported on silica in the process of catalyst preparation have been studied in the literature [28,29]. The authors of [28] have studied Cu-containing silica after calcination and exposure to air whose EPR spectrum showed features corresponding to g_ǁ_ = 2.382 and A_ǁ_ = 136 × 10^−4^ cm^−1^ (123.6 G). These values are very similar to the results obtained in this study. The same authors attributed the paramagnetic copper complex to a Cu(II) ion coordinated to four water molecules and two –O^−^ singly charged oxygen atoms on the silica surface. On the other hand, Gervasini and coworkers [29] have suggested that the copper complex in Cu/SiO_2_ catalyst should be identified as Cu coordinated to two neutral –OH groups on the silica surface, in addition to four water molecules. The overall charge of this complex is +2.

**Figure 4:**
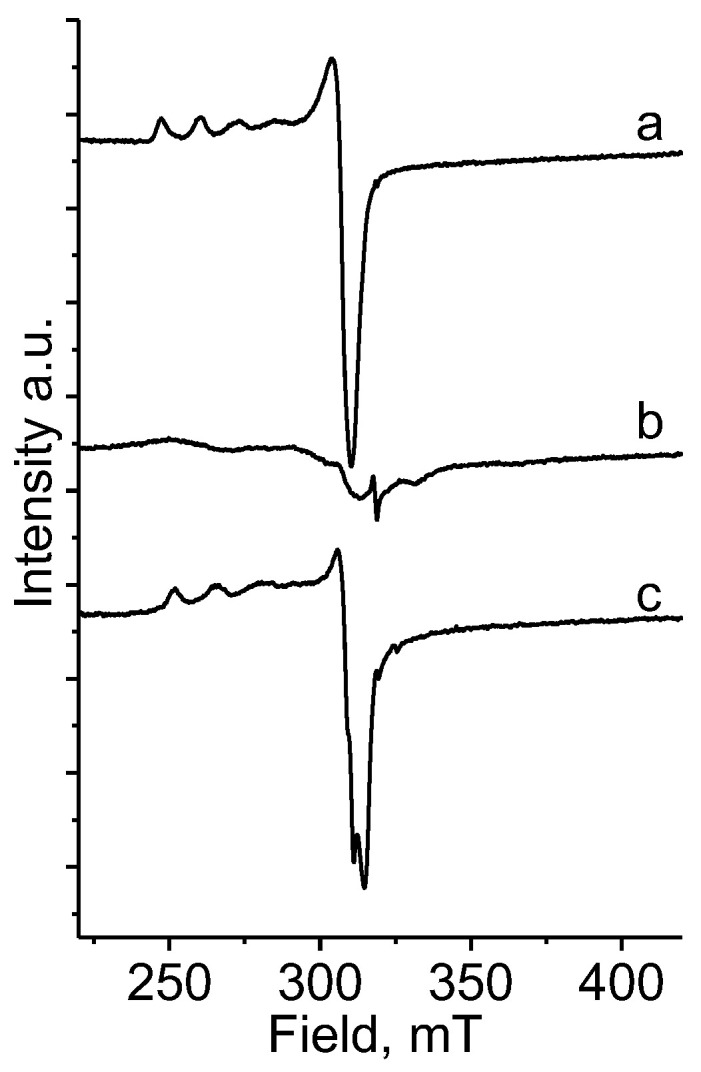
EPR spectra of Cu-SBA-15 fresh sample (a), after evacuation at 623 K (b) and after adsorption of NO (c). Spectra recorded at 77 K.

Interestingly, in this study, the evacuation of Cu-SBA-15 at 623 K in a vacuum (4 × 10^−3^ mbar) caused a significant reduction in the intensity of the EPR signal originating from the paramagnetic copper complex (Figure 4). The EPR signal from copper in the octahedral environment disappeared completely. There was also a sharp peak at g = 1.998 with width ΔHpp = 1.1 mT, which can be attributed to the organic radical derived from residuals of the template used in the synthesis of SBA-15. In the parallel region, after evacuation at 623 K, hfs is poorly resolved and the intensity of the EPR signal is much weaker, similar as in the perpendicular region.

It is a known phenomenon for Cu cations in zeolites, that evacuation at a temperature higher than 473 K leads to autoreduction of Cu^2+^ to Cu^+^ [30]. Considering such a possibility, NO was used as a probe molecule for the detection of diamagnetic Cu^+^ cations.

The EPR spectra of the initial sample and the same sample after vacuum treatment as well as after NO adsorption are shown in Figure 4 and the signal parameters are presented in Table 2.

NO adsorption caused a significant increase in the intensity of the EPR signal. NO oxidizes reduced copper and forms paramagnetic complexes with it. However, it is not a typical NO complex with diamagnetic Cu^+^ cations, observed by many researchers (e.g., [31,32]). Probably after evacuation at 623 K, another type of diamagnetic copper species was formed and led to the formation of the paramagnetic complex revealed by the EPR signal in spectrum c in Figure 4. The increase in the signal intensity after NO adsorption on the evacuated catalyst and changes in the spectrum parameters are most probably related to the presence of Cu^2+^O^−^NO or other Cu^δ+^NO (1 < δ <2) paramagnetic complex. Cu^2+^O^−^ species produced by autoreduction of copper cations in a vacuum are EPR silent similar to copper with a residual charge Cu^δ+^, but with NO they form paramagnetic complexes [30,33]. The spin–Hamiltonian parameters for Cu-SBA-15 after adsorption of NO, i.e., the values of the tensor g_ǁ_ = 2.32 and hyperfine splitting A_ǁ_ = 150.7 G, are characteristic of copper in an environment of square pyramidal symmetry. A very similar spectrum of the Cu-NbZSM-5 zeolite was presented in [33] and it was assigned to Cu^2+^O^−^NO or other Cu^δ+^NO paramagnetic species.

It is important to point out that activation of the catalysts before the catalytic reaction (ethanol dehydrogenation) was performed at 623 K under inert gas flow. Such conditions are very similar to the evacuation. Therefore, one can conclude that the copper species identified for Cu-SBA-15 by EPR (Cu^δ+^ (1 < δ < 2)) are present on the catalyst surface before the ethanol transformation reaction.

### 2.3. Acid-Base Properties of Catalysts

Identification of the nature and number of acid sites on the catalyst surface was achieved on the basis of a pyridine adsorption/desorption study combined with infrared spectroscopy measurements. Typically, pyridine coordinatively bonded to Lewis acid sites (LAS) gives rise to symmetric (~1450 cm^−1^) and antisymmetric (~1605 cm^−1^) vibrations in the chemisorbed pyridine molecule [34,35], whereas the bands at 1446 cm^−1^ and 1597 cm^−1^ originate from the vibration in pyridine hydrogen bonded to silanol groups in SBA-15 or to weak BrØnsted acid sites (BAS) [10,36]. Pyridine chemisorbed on stronger BAS forms pyridine cations which give characteristic bands at ~1545 cm^−1^ and 1637 cm^−1^ coming from the vibrations in pyridine cations. As shown in Supplementary Figure S3, the latter bands are not observed in the spectra of Cu-SBA-15, MnSBA-15, and Cu-MnSBA-15 after pyridine adsorption showing the lack of BAS on the surface of the catalyst.

The number of LAS on the catalyst’s surface, calculated from the amount of pyridine adsorbed at 523 K (and desorbed at the same temperature) and after desorption at 573 K, are shown in Table 3. The strength of LAS can be estimated from the amount of pyridine desorbed at 573 K in relation to the amount of pyridine present on the surface after adsorption/desorption at 523 K. In MnSBA-15 sample, a small number of LAS was detected. They originate from manganese cationic species, so their low number is in line with a small amount (0.5 wt.%) of manganese species in the material. The LAS in MnSBA-15 are relatively weak because 48% of pyridine adsorbed/desorbed at 523 K was desorbed by the evacuation at 573 K. Copper loading (2.2 wt.%) on MnSBA-15 resulted not only in a significant increase in the LAS number but also in a substantial increase in their strength (19% of pyridine molecules adsorbed on LAS after adsorption/desorption at 523 K was desorbed upon evacuation at 573 K). The number of LAS in Cu-SBA-15 was lower than that in Cu-MnSBA-15 because of a lower copper loading (2.0 wt.%) and the lack of manganese species also playing a role of LAS. Interestingly, the LAS in Cu-SBA-15 were much weaker (37% of pyridine was desorbed at 573 K) than in Cu-MnSBA-15, suggesting the enhancement of LAS strength by the synergistic interaction between copper and manganese species.

The acid/base properties of the samples were also studied using the test reaction of 2-propanol decomposition [37]. The activity and selectivity of this reaction are shown in Table 4. A comparison of the results for the supports (SBA-15 and MnSBA-15–entries 1,3) indicates the generation of acid centers on the surface of silica after manganese introduction (deduced from propene formation and an increase in 2-propanol conversion from 1% to 8%). The selectivity to propene, formed on acid sites of above 90%, was achieved for this sample. For MnSBA-15 also some activity of basic centers (6% selectivity to acetone) and acid-base pairs (3% selectivity to the diisopropyl ether) was evidenced.

Modification of both supports with copper led to the increase in alcohol conversion (17% for Cu-SBA-15 and 15% for Cu-MnSBA-15) and changed the surface properties of the catalysts due to the generation of additional basic/redox centers (increase in selectivity to acetone, formed on basic/redox sites [37]). Moreover, the increase in Lewis acid-base pairs on Cu-SBA-15 can be deduced from the significant increase in selectivity to diisopropyl ether (Table 4, entry 2). It is worth noting that the selectivity to acetone was the highest for Cu-MnSBA-15, whereas, for Cu-SBA-15, the highest selectivity to the ether was observed. For both copper-containing materials (Table 4, entries 2 and 4) the dominating product was propene (66% and 59% selectivity on Cu-SBA-15 and Cu-MnSBA-15, respectively) formed via dehydration of alcohol on Lewis acid sites of the catalysts. Thus, one can conclude that LAS are present on Cu-SBA-15 catalyst surface as: (i) isolated acid centers responsible for propene formation, and (ii) in the close neighborhood of Lewis basic sites (LBS) forming LAS-BAS pairs (CuO) controlling ether generation. Moreover, Cu-SBA-15 has isolated LBS taking part in the dehydrogenation of 2-propanol to acetone. In the presence of Cu-MnSBA-15, mainly propene and acetone appeared in the reaction products and ether formation was negligible (Table 3, entry 4), which was evidence of the reduction of LAS–LBS pairs activity. Most probably it was the effect of interaction between the framework manganese and copper (CuO) species whose presence was postulated on the basis of the Uv-vis study described above.

Interestingly, the pretreatment of Cu-SBA-15 catalyst in oxygen flow significantly changed the selectivity of the reaction. The selectivity to propene decreased (from 66 to 45%), whereas an increase in the selectivity to acetone was observed (from 14 to 55%). It indicates the blockage of acidic sites (LAS) by oxygen and the generation of basic sites (LBS) as a result of oxygen adsorption on copper cationic species.

To check if BrØnsted basic sites (BBS) are present on the surface of the catalyst, another test reaction was performed–cyclization and dehydration of 2,5-hexanedione. In this reaction methylcyclopentenone (MCP) is formed with the participation of BBS, whereas BAS are responsible for the production of 2,5-dimethylfuran (DMF) [38]. If the basic BrØnsted centers dominate on the catalyst surface, the selectivity ratio MCP/DMF is >1, while MCP/DMF < 1 indicates that there are more BrØnsted acidic sites on the surface.

The results shown in Table 5 indicate the basic character of SBA-15, MnSBA-15, and Cu-MnSBA-15 samples (high selectivity to MCP, MCP/DMF > 1). However, it is worth noting that the activity of SBA-15 support was very low and it increased after the modification with Mn (from 2 to 10%) and that MnSBA-15 was characterized by the highest MCP/DMF ratio. It suggests the generation of BBS after the introduction of manganese into the SBA-15 structure. Moreover, it is clear that the modification of MnSBA-15 with copper increased the activity, but decreased the basicity of the surface (the decrease in MCF/DMF ratio from 19.0 to 3.3). Cu-SBA-15 (without manganese) showed lower activity than Cu-MnSBA-15, and acidic character (high selectivity to DMF, MCP/DMF < 1). As the above described results of pyridine adsorption combined with those of the FTIR study did not show the presence of BAS, we postulate that in the first step of the reaction, MCP is formed with the participation of BBS and this process is accompanied by water formation. At the high reaction temperature (623 K) water vapor generates BAS on Lewis acidic sites and BAS cause the transformation of 2,5-hexanedione to DMF. Thus, on the basis of the test on BrØnsted acidity-basicity, we postulate the presence of only BBS on the catalyst surface after activation.

### 2.4. Ethanol Dehydrogenation–FTIR Study in Flow System

To get insight into the species formed on the surface of copper-containing catalysts and the gas phase composition after ethanol adsorption and dehydrogenation as well as interaction with oxygen, an FTIR study of ethanol (EtOH) transformation (without and with oxygen) in the flow system was performed. The study was performed with the use of all catalysts and supports by measurements of FTIR spectra at the intervals of 5 min (throughout 120 min total) during the reaction carried out at 473 K or 593 K and during desorption (60 min) at the same temperature. As shown in Supplementary Figure S4, starting from the 45th min of the reaction at 593 K, the intensity of the band at 1746 cm^−1^ (coming from C=O vibrations in aldehyde [39,40]–the reaction product) became stable. Therefore, all comparative studies were performed based on the spectra obtained after 45 min on stream.

#### 2.4.1. The Effect of the Reaction Temperature

**Figure 5:**
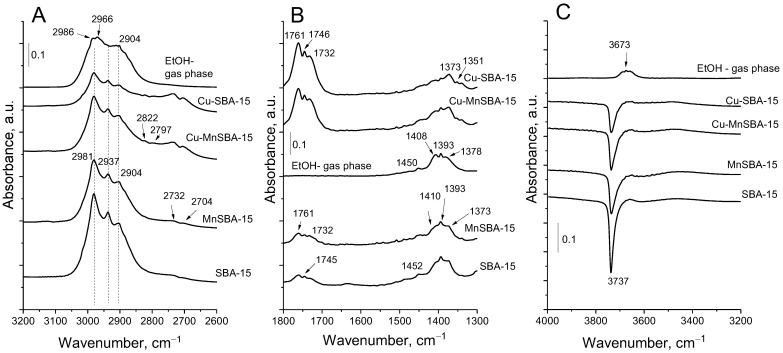
FTIR spectra recorded in ethanol flow at 593 K after 45 min of the dehydrogenation reaction for SBA-15, MnSBA-15, Cu-SBA-15, and Cu-MnSBA-15 materials: (**A**) 3200–2600 cm^−1^ range; (**B**) 1800–1300 cm^−1^ range; (**C**) 4000–3200 cm^−1^ range.

Figure 5 shows the IR subtracted spectra of copper SBA-15 catalysts and their supports after activation in Ar at 623 K followed by EtOH flow at 593 K after 45 min of the dehydrogenation reaction (the sample in the reactor was in a stream of EtOH + Ar). The spectra are compared with the spectrum of the gas phase of alcohol recorded at 593 K, which allows us to identify the species formed during the reaction (chemisorbed on the catalyst surface and in the gas phase). The characteristic bands assigned to ethanol in the gas phase have maxima at ca. 3680 cm^–1^ corresponding to non-bonded valence O–H vibrations (free or unassociated hydroxyl groups (νOH)), maxima in the range 2800–3000 cm^–1^ from valent C-H bonds (CH_3_ groups (νCH_3_) and CH_2_ groups (νCH_2_)) and in the range 1400–1300 cm^–1^ characteristic of skeleton vibrations of the C–O–H groups [41].

The bands from ethanol in the gas phase (2986, 2966, 2904, 1408, 1393, 1378 cm^−1^) are also visible in the spectra of all studied catalysts after 45 min of the ethanol transformation at 593 K, but the two bands at 2986 cm^–1^ and 2966 cm^–1^ are combined to one with a maximum at 2981 cm^–1^ suggesting a kind of stabilization of ethanol molecules on the catalyst surface (molecularly adsorbed alcohol–Figure 5A [42,43]. The bands at 2981 cm^–1^ and 2904 cm^–1^ cover the region characteristic of C-H vibration in both gas EtOH and alcohol molecularly chemisorbed on the solid surface. They also overlap the bands coming from the characteristic C-H vibrations in acetaldehyde [43]. It is important to note that in the range of 2850–3000 cm^–1^, a new band at 2937 cm^–1^ (not observed in the gas ethanol) appears in the spectra scanned during the reaction on supports and copper catalysts. There are no significant differences in this region in the spectra for all samples. Thus, three bands dominate in the spectra of all catalysts contacted with EtOH. These are the bands at 2981, 2937, and 2904 cm^−1^. On the basis of the literature data, the band at 2937 cm^−1^ (absent in the spectrum of gas EtOH) should be assigned to ethoxy groups [42]. It indicates that ethanol was dissociatively chemisorbed on all SBA-15 materials in the form of ethoxy species which were also responsible for the bands at ca. 2980 and 2900 cm^−1^, besides the band at 2937 cm^−1^. Thus, the presence of two types of adsorbed ethanol species can be concluded: molecularly and dissociatively adsorbed alcohol. It is important to add that in the region 3000–2850 cm^−1^ the bands characteristic of gas phase/molecularly adsorbed/dissociatively adsorbed ethanol are much more intense for both supports and Cu-MnSBA-15 than for Cu-SBA-15, indicating more efficient transformation of chemisorbed ethanol to acetaldehyde on Cu-SBA-15.

Moreover, ethanol flow at 593 K through the catalyst wafer led to the appearance of a broad band at ca. 3500–3300 cm^−1^ typical of hydrogen bonding to hydroxyls (Figure 5C). The negative band at ca. 3740 cm^−1^ typical of silanol groups (Si–OH) in the SBA-15 matrix increases in intensity in the subtracted spectra after EtOH admission. It suggests the formation of hydrogen bonded species (that could be either ethanol or acetaldehyde hydrogen bonded to silanol groups). It is important to add that, as shown in Supplementary Figure S5, the intensity of the band at 3737 cm^−1^ from the vibrations in silanol groups after activation of the samples is much smaller on the copper-containing materials than on the supports. A lower intensity of this band was observed in the spectrum of MnSBA-15. This observation indicates that the modification of SBA-15 with manganese and copper species is accompanied by the partial consumption of silanol groups. It is the reason why the intensities of negative bands presented in Figure 5C are different for different samples, but their changes are considered in relation to the starting number of silanol groups in the materials. Supplementary Figure S5 also shows that in the spectra of all SBA-15 samples modified with Mn and Cu, the band at 3737 cm^−1^ from silanol groups has a tail from the side of lower wavenumbers, covering the signals from the other OH groups, including BrØnsted basic centers, identified by the cyclization and dehydration of 2,5-hexanedione.

**Figure 6:**
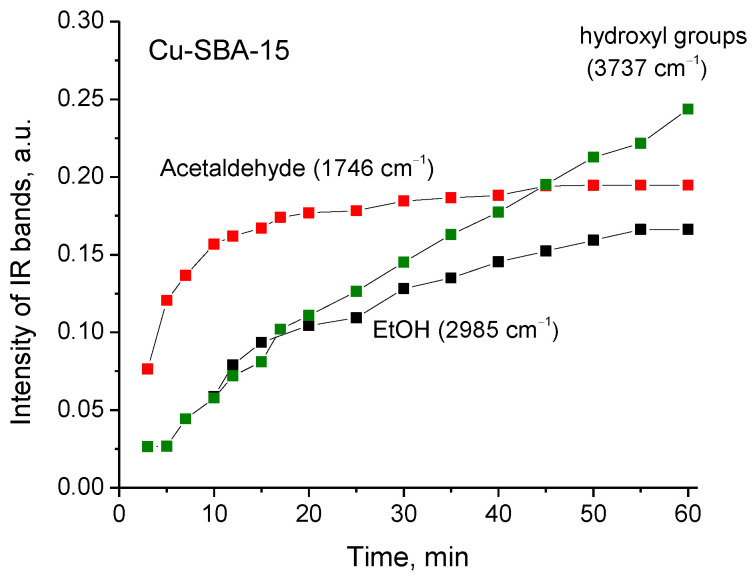
Changes in the intensity of IR bands in the spectra recorded for Cu-SBA-15 catalyst in ethanol flow at 593 K during 60 min: EtOH (black), acetaldehyde (red), and consumption of hydroxyl groups (green).

As shown in Figure 6, at the beginning of the reaction (up to 15 min) the curves illustrating an increase in the intensity of the bands from gas/molecularly/dissociatively adsorbed/hydrogen bonded ethanol (3000–2850 cm^−1^ region) and an increase in the consumption of silanol groups, exhibit the same slope suggesting the participation of hydroxyls in the adsorption of ethanol. With the time on stream, the slopes of both curves differ significantly demonstrating the participation of acetaldehyde chemisorption in the consumption of Si-OH.

To sum up the discussion about the origin of the bands appearing in the region 3000–2850 cm^−1^, it has been established that the two bands at 2981 and 2904 cm^−1^ are characteristic of C-H vibrations in ethanol (gas, hydrogen bonded to OH groups, molecularly chemisorbed and dissociatively chemisorbed in the form of ethoxy species) and acetaldehyde. The band at 2937 cm^−1^ is concluded as characteristic of ethoxy species.

The most spectacular differences in the IR spectra of SBA-15 supports (SBA-15 and MnSBA-15) and copper catalysts (in comparison to that of ethanol in the gas phase) are observed in the regions of C=O vibrations (1800–1650 cm^–1^)–Figure 5B and H-C=O vibrations (2850–2700 cm^–1^)–Figure 5A. Three bands at 1761, 1746, and 1732 cm^–1^ appear in the spectra during ethanol flow in the presence of the catalysts studied. These bands are accompanied by four others at 2822, 2797, 2732, and 2704 cm^−1^ assigned to C-H vibrations in H-C=O of aldehyde adsorbed on Lewis acidic centers (the pair at 2822 and 2797 cm^−1^ from symmetric and asymmetric stretching vibrations [1,43]) and hydrogen bonded acetaldehyde (the pair at 2732 and 2704 cm^−1^ from symmetric and asymmetric stretching vibrations [43]). The band at 1746 cm^–1^ that was previously observed in the spectra of Co-Y zeolites after interaction with EtOH [40] was assigned to C=O stretching vibrations in the reaction product–acetaldehyde. It should be noted that the intensity of the bands characteristic of acetaldehyde is significantly lower in the spectra of the supports (the bands at 2822 and 2797 cm^–1^ are visible only for copper catalysts). It means that acetaldehyde is chemisorbed (dissociatively) with the participation of Lewis acidic sites (LAS) coming mainly from copper species (isolated copper cations or CuO) and basic sites. The highest production of acetaldehyde (the bands in the region 1800–1650 cm^–1^ of C=O vibrations) was observed for the Cu-SBA-15 sample, i.e., the material having a lower number of weaker LAS (calculated from pyridine adsorption) and a lower basicity (both BrØnsted and Lewis basicity deduced from 2,5-hexanedione and 2-propanol transformations) than Cu-MnSBA-15 sample. Interestingly, the amount of acetaldehyde (the bands in the region of 1800–1650 cm^–1^) was much higher in the products of the reaction in the presence of Cu-SBA-15 than Cu-MnSBA-15, whereas dissociatively/molecularly adsorbed/hydrogen bonded/gas ethanol was in a higher amount (the bands in the region of 3000–2850 cm^–1^) for Cu-MnSBA-15. This observation suggests the importance of the relatively low strength of pairs LAS and basic sites for the effective acetaldehyde production at 593 K. Because of copper-manganese synergistic interaction in Cu-MnSBA-15, the strength of LAS increased and ethoxy species became too strongly held on LAS to be further transformed to acetaldehyde.

The spectra in the range of 1500–1300 cm^−1^ (Figure 5B) cannot be used as fingerprints for analysis of ethanol and acetaldehyde chemisorption because this region displays the bands from gas ethanol (1450, 1408, 1393, and 1378 cm^−1^) as well as from C-H asymmetric vibration of CH_3_ in ethoxy species or molecularly adsorbed acetaldehyde (1452 cm^−1^) [42,43] and C-H bending symmetric vibration in an acetoxy methyl group (1373 cm^−1^) [42]. Therefore, it is difficult to assign the observed bands. However, changes in the spectrum profile in this region are well visible if copper-containing catalysts are used, especially for Cu-SBA-15 material. The spectrum of this sample shows the highest intensity of the band at 1373 cm^−1^ (C-H bending symmetric in acetoxy species [42]) characteristic of chemisorbed acetaldehyde in contrast to the spectra obtained if supports only were used as catalysts in the reaction. It is important to underline that the vibration band characteristic of ethene, at 1508 cm^−1^ [40] (the product of the side reaction- dehydration of ethanol) was not detected in the infrared spectra. Thus, the studied materials did not contain acidic sites strong enough to direct the reaction towards the undesired dehydration pathway.

**Figure 7:**
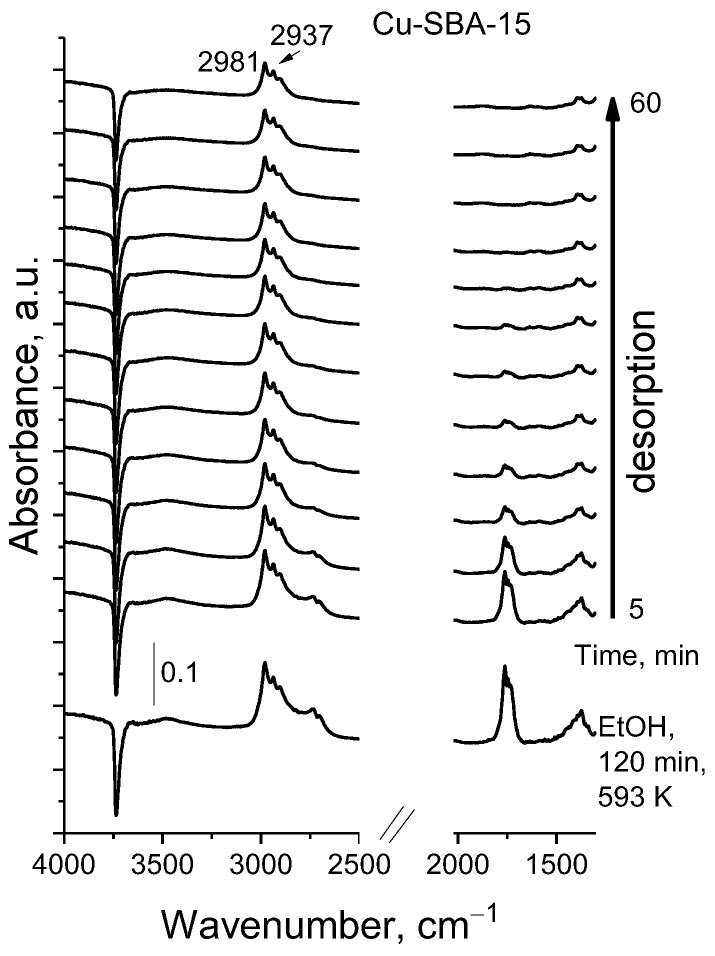
FTIR spectra (4000–1300 cm^−1^ range) recorded in argon flow at 593 K for 60 min after 120 min of ethanol dehydrogenation reaction at 593 K for Cu-SBA-15.

The desorption experiments performed at 593 K during 60 min in argon flow after the reaction of ethanol transformation at the same temperature provided a deeper insight into the species formed during the reaction, especially their stability. The spectra presented in Figure 7 illustrate the changes occurring during the desorption in the experiment with the use of a Cu-SBA-15 sample.

After 30 min of the desorption, the intensity of the band at 2981 cm^−1^ assigned to hydrogen bonded/molecularly/dissociatively adsorbed ethanol and overlapped with the band originating from gas ethanol, dropping to ca. 60% of the preliminary one. Further desorption for 30 min (a total of 60 min) did not change the intensity of this band. The same phenomenon was observed for the band at 2937 cm^−1^ characteristic of ethoxy species. It means that the bands in this region originate partly from weakly adsorbed or gas ethanol (ca. 40%) and partly from the strongly chemisorbed species (ethoxy and/or acetoxy). Interestingly, the intensity of the bands in the regions 1800–1650 cm^–1^ and 2850–2700 cm^–1^ systematically decreased and after 30 min the bands disappeared (removal of gas and adsorbed acetaldehyde). These changes in the infrared spectra were accompanied by the partial rebuilding of hydroxyl groups (ca. 25% silanol groups were recovered). The fact that the bands in the region 1500–1300 cm^−1^ did not disappear, but only decreased and changed their shape, indicates that they originate from both, gas ethanol and relatively strongly chemisorbed ethanol/acetaldehyde.

To sum up, the results obtained in the reaction of ethanol at 593 K, three types of adsorbed ethanol species were identified: (i) molecularly adsorbed, (ii) hydrogen bonded, and iii) dissociatively adsorbed in the form of ethoxy species. The latter exists in two types; (i) ethoxy species chemisorbed on LAS (weakly held and desorbed after ca 30 min) and (ii) stable ethoxy included to the silica structure by the interaction of silanol groups with EtOH as postulated for Ag/SiO_2_ in [9].

**Figure 8:**
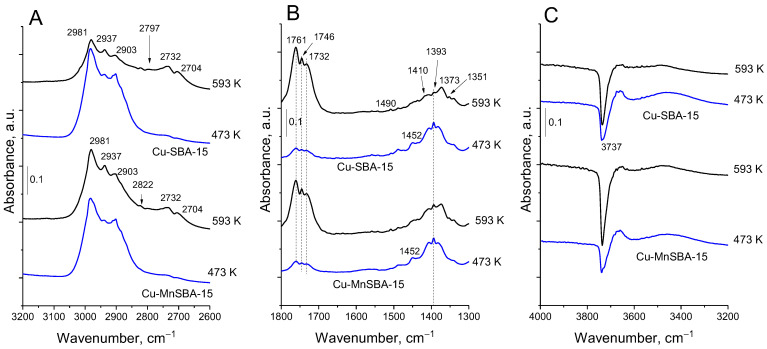
FTIR spectra recorded in ethanol flow at 473 K (blue) and 593 K (black) after 45 min of the dehydrogenation reaction for Cu-SBA-15 and Cu-MnSBA-15 materials: (**A**) 3200–2600 cm^−1^ range; (**B**) 1800–1300 cm^−1^ range; (**C**) 4000–3200 cm^−1^ range.

A decrease in the reaction temperature to 473 K significantly reduced the activity of the samples. Figure 8 shows a comparison of the IR subtracted spectra of copper catalysts (Cu-SBA-15 and Cu-MnSB-15) during EtOH flow at 423 K and 593 K after 45 min of the dehydrogenation reaction (EtOH + Ar flow). In the range characteristic of the C=O vibrations (1800–1650 cm^–1^), the bands assigned to acetaldehyde appeared at 1761, 1746, and 1732 cm^–1^ in the spectra of the catalysts at both temperatures (Figure 8B). However, the intensity of these bands for the catalysts treated with alcohol at 473 K was much lower than that observed at 593 K. Moreover, the bands from the aldehyde adsorbed on LAS at 2822, 2797 cm^−1^ (C-H vibrations in H-C=O) were well pronounced only in the spectra of the catalysts recorded during the reaction at 593 K C (Figure 8A). The bands at 2732 and 2704 cm^−1^ (C-H vibration in acetaldehyde hydrogen bonded) were slightly visible. It indicates that the dehydrogenation of ethanol to acetaldehyde takes place to a minimal extent at 473 K and a higher temperature is required for alcohol transformation by the dehydrogenation process.

The band at 2937 cm^−1^ (Figure 8A), characteristic of ethoxy species, is also visible in the spectra of all catalysts treated with ethanol at 473 K, which confirms the dissociative adsorption of ethanol chemisorbed on copper-containing SBA-15. Comparison with the spectra at the higher reaction temperature (593 K) shows that the difference again appears in the intensity of the bands in the region of 2800–3000 cm^−1^. The intensities of the bands at 2937 cm^−1^ (ethoxy groups) and 2981, 2904 cm^−1^ (gas phase/molecularly/dissociatively adsorbed/hydrogen bonded ethanol) are much lower for the reaction performed at 593 K. Participation of hydrogen bonded ethanol species in the chemisorbed form during the reaction at 473 K is confirmed by a higher intensity of the broad band at ca. 3450 cm^−1^ than the corresponding one for the reaction at 593 K. Moreover, the spectra recorded during desorption in the experiment performed at 593 K (Supplementary Figure S6) indicate that after 30 min of the process, ca. 45% of the species characterized by the bands in the region 2800–3000 cm^−1^ are still present and this percentage was stable during the following 30 min of the desorption. It confirms the incorporation of stable dissociatively chemisorbed ethanol into the silica skeleton.

To sum up, at 473 K the dehydrogenation of ethanol to acetaldehyde is negligible, whereas, dissociatively chemisorbed ethanol is relatively stable. Moreover, there are no significant differences in the spectra recorded during the reaction at 473 K over Cu-SBA-15 and Cu-MnSBA-15 in contrast to the reaction performed at 593 K in which Cu-SBA-15 was much more active. The reaction at 473 K was applied for further study of the effect of oxygen admission to the reaction mixture.

#### 2.4.2. The Effect of Oxygen in the Reaction Mixture

**Figure 9:**
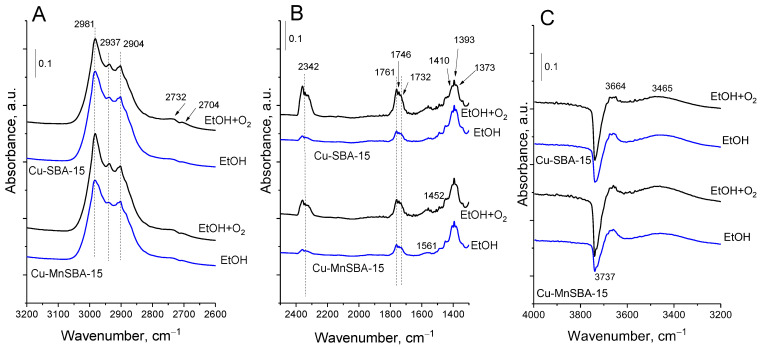
A comparison of the FTIR spectra recorded in ethanol (blue) and ethanol with oxygen (black) flow at 473 K after 45 min of the dehydrogenation reaction for Cu-SBA-15 and Cu-MnSBA-15 materials: (**A**) 3200–2600 cm^−1^ range; (**B**) 2500–1300 cm^−1^ range; (**C**) 4000–3200 cm^−1^ range.

**Figure 10:**
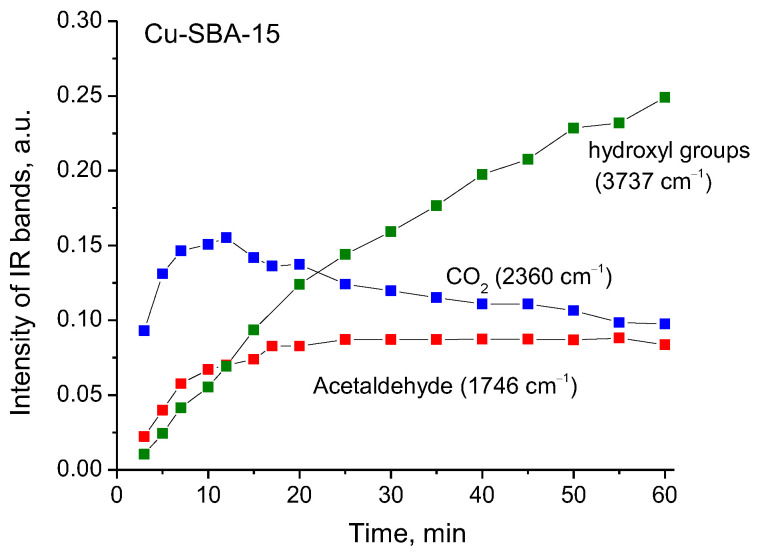
Changes in the intensity of IR bands in the spectra recorded for Cu-SBA-15 catalyst in ethanol and oxygen flow at 473 K during 60 min: acetaldehyde (red), CO_2_ (blue), and consumption of hydroxyl groups (green).

In order to check if the introduction of oxygen to the ethanol flow changes the activity and selectivity of copper and copper-manganese catalysts based on SBA-15 supports in ethanol to acetaldehyde dehydrogenation at 473 K, the experiments in EtOH + O_2_ flow were performed. The results are shown in Figure 9. A significant increase in the intensity of the bands characteristic of acetaldehyde (1800–1650 cm^–1^ and 2850–2700 cm^–1^ regions) was observed after the addition of oxygen. This effect was more pronounced for Cu-SBA-15 than for Cu-MnSBA-15, confirming the negative role of copper-manganese synergistic interaction reducing the activity of LAS-LBS pairs. There was no significant difference in the region of 3000–2850 cm^−1^ (Figure 9A) in the spectra of all copper-containing samples in the flow of ethanol and the mixture of ethanol and oxygen. In this region, the bands characteristic of the gas phase and dissociatively/molecularly adsorbed/hydrogen bonded ethanol occur. Thus, the presence of oxygen in the gas mixture does not increase the adsorption of alcohol but enhances the rate of ethanol dehydrogenation to acetaldehyde. Interestingly, the oxygen admission to the reagents feeds led to an increase in the consumption of silanol groups (Figure 9C) which was accompanied by an increase in the intensity of 2732 and 2704 cm^−1^ bands (Figure 9A) coming from acetaldehyde hydrogen bonded species. The slopes of the curves illustrating the Si-OH consumption and EtOH adsorbed species are almost the same. The positive effect of oxygen on the alcohol dehydrogenation reaction resulted in the formation of a larger amount of gas and molecularly adsorbed (via hydrogen bonding) acetaldehyde than in the reaction without oxygen in the feed. However, the selectivity to acetaldehyde was diminished by the formation of CO_2_ (the bands at ca. 2340 cm^−1^–Figure 9B). Figure 10 shows that at the beginning of the reaction, the main reaction route was total oxidation to CO_2_ which slightly and systematically decreases after 15 min of the reaction, whereas acetaldehyde formation increased from the beginning of the reaction, and after ca. 25 min the amount of acetaldehyde became stable.

**Figure 11:**
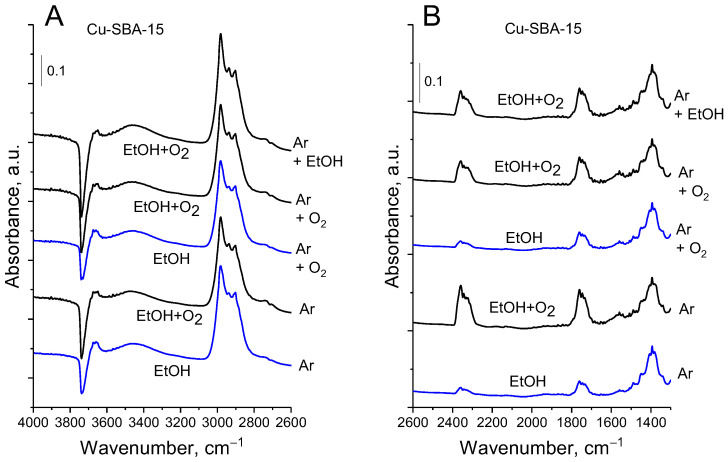
A comparison of the FTIR spectra recorded in ethanol (blue) and ethanol with oxygen (black) flow at 473 K after 45 min of the reaction for Cu-SBA-15 activated in Ar or Ar + O_2_ or Ar + EtOH mixture: (**A**) 4000–2600 cm^−1^ range; (**B**) 2600–1300 cm^−1^ range.

To get a deeper insight into the role of oxygen in the improvement of acetaldehyde formation from ethanol at 473 K, two additional experiments were performed for Cu-SBA-15 (the most active catalyst). In both experiments, the catalysts were activated in oxygen flow at 473 K before the introduction of (i) ethanol and oxygen mixture or (ii) only ethanol. As follows from Figure 11 and Supplementary Figure S7 showing the spectra of Cu-SBA-15 after activation in Ar and after activation in Ar + O_2_, for 45 min of the reaction in EtOH + O_2_ flow, the intensity of the bands assigned to acetaldehyde (1800–1650 cm^–1^) and CO_2_ decreases in comparison to that of the same bands in the spectra of the catalyst activated only in Ar. This observation indicates the blockage of the active centers responsible for product formation. In contrast, the activation of the catalyst in oxygen flow did not change the infrared spectra recorded in the ethanol flow only (without admission of oxygen during the reaction)–Figure 11 and Supplementary Figure S8. It means that oxygen adsorption on the catalyst surface does not increase its activity in ethanol dehydrogenation to acetaldehyde. Interestingly, there are no changes in the FTIR spectra in the region 3000–2850 cm^−1^ for both EtOH and EtOH + O_2_ reagents after catalysts activation with oxygen. On the other hand, the preadsorption of ethanol before EtOH + O_2_ flow significantly increases the consumption of hydroxyl groups and the intensities of the bands characteristic of gas/molecularly/dissociatively adsorbed/hydrogen bonded ethanol (3000–2850 cm^−1^) but decreases CO_2_ and acetaldehyde production, the former much more (by 40%) than aldehyde (by 20%). Thus, the blockage of LAS and/or hydroxyls by ethanol chemisorption decreases the catalyst activity in ethanol dehydrogenation to acetaldehyde.

**Figure 12:**
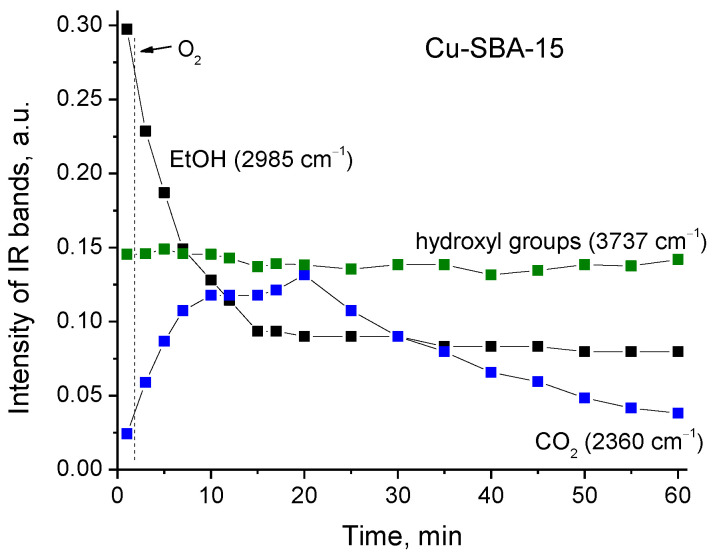
Changes in the intensity of the IR bands in the spectra recorded after pretreatment of Cu-SBA-15 catalyst with ethanol at 473 K for 45 min and oxygen flow included for the following 60 min: EtOH (black), CO_2_ (blue), and consumption of hydroxyl groups (green).

In the experiment with EtOH flow at 473 K, cutting off ethanol flow after 60 min and introducing oxygen flow for the following 60 min, the obtained spectra showed the formation of CO_2_ accompanied by a significant decrease in the number of ethanol species and no changes in the hydroxyl region (Figure 12). Interestingly, after 15 min of oxygen flow, the intensity of the infrared band from the ethanol adsorbed species stabilized and CO_2_ was systematically desorbed. It means that a part of ethanol species (ethoxy introduced into a silica skeleton via the interaction between –Si-OH and EtOH) did not participate in the total oxidation of alcohol.

To sum up, the admission of oxygen to the reaction mixture significantly increased the activity of the catalysts in ethanol dehydrogenation to acetaldehyde performed at 473 K. However, the pretreatment of the catalysts with oxygen as well as with ethanol flow before the reaction (EtOH + O_2_) decreased their activity in dehydrogenation of ethanol. Thus, chemisorption of both, EtOH and O_2_ blocked the centers required for effective ethanol dehydrogenation in the presence of oxygen. The adsorption of oxygen or EtOH before the reaction blocked LAS, as deduced from the lack of IR bands from acetaldehyde (2822 and 2797 cm^−1^) chemisorbed on LAS in both, the reactions of EtOH and EtOH + O_2_ and a decrease in the acidic site’s activity with simultaneous increase in the basic (LBS) activity in 2-propanol transformation. It means that LAS are important in the dehydrogenation reaction pathway. However, as underlined in the Introduction, pairs of basic and acidic centers are involved in ethanol dehydrogenation. Nevertheless, as shown by the above described results, the increase in basicity by the admission of manganese to the support of copper catalyst or by the preadsorption of oxygen not only did not enhance the activity of Cu-MnSBA-15 but even partially reduced it by chemisorption of oxygen on LAS.

The reaction pathways’ dependence on the reaction temperature and the presence of oxygen are discussed below.

#### 2.4.3. Discussion

Based on UV-vis and EPR studies, Cu^2+^ isolated ions and CuO have been identified in fresh Cu-SBA-15 and Cu-MnSBA-15 catalysts. The analysis of the results of XPS indicated the presence of Cu^δ+^ (1 < δ < 2) isolated ions in the Cu-SBA-15 sample after evacuation at r.t., whereas in Cu-MnSBA-15 additionally Cu^2+^ in copper oxide(II) were found. What is important, Cu^δ+^ (1 < δ < 2) ions were recognized by EPR studies as the main species in Cu-SBA-15 evacuated at 623 K. The same copper species should be present on the surface of the Cu-SBA-15 catalyst activated at 623 K in argon flow before ethanol transformation.

On the basis of the literature data [6,9,44], two possible routes for the dehydrogenation of ethanol to acetaldehyde can be proposed, depending on the catalysts used: (i) ethoxy or ethoxide intermediate way and (ii) H-bonded complex route. In the latter route, silanol groups from the support participate in the formation of this complex, and the presence of species able to abstract protons (such as metallic silver) and form hydrogen molecules and acetaldehyde is needed. Although the presence of hydrogen bonded species was inferred from the IR spectra (a broad band at ca. 3400 cm^−1^), their amount was negligible in the reaction performed at 593 K, i.e., at the temperature in which the catalyst activity was significantly higher than at 473 K. Moreover, although acetaldehyde formation was stabilized after 45 min of the reaction (Figure 6) and the chemisorption of ethanol molecules was stabilized starting from 55 min on stream, the consumption of hydroxyl groups increased linearly, so that the same slope was maintained up to 60 min of the reaction.

**Scheme 1:**
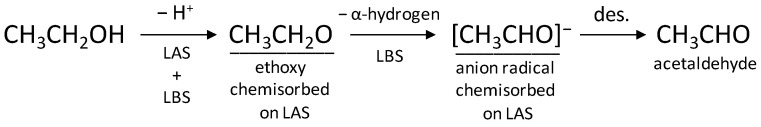
Dehydrogenation of ethanol at 593 K–ethoxy reaction pathway.

Combining these results with the evidence of the relationship between Lewis acidity and the activity of the catalysts, we propose the domination of the ethoxy intermediate route for ethanol dehydrogenation on copper-containing catalysts used in this work. For the reaction performed at 593 K in the first step of the reaction, dissociative adsorption of ethanol takes place on pairs of LAS and LBS with balanced strength via abstraction of a proton from OH group in alcohol, and surface ethoxy species are formed (Scheme 1). In the next step, the elimination of α-hydrogen from methylene group (CH_2_) in ethoxy species occurs with the participation of LBS, and chemisorbed anion radicals [CH_3_CHO]^−^ are formed, which are adsorbed on LAS and are relatively easily transformed to acetaldehyde and desorbed to the gas phase.

**Scheme 2:**
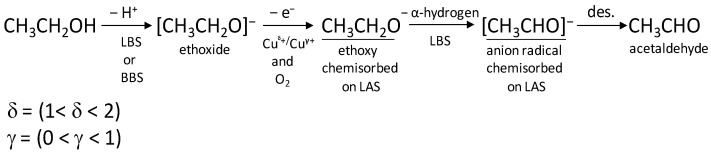
Dehydrogenation of ethanol in the presence of oxygen at 473 K-ethoxide reaction pathway.

At the lower reaction temperature (473 K) the LAS-LBS pairs seem to be not strong enough for efficient dehydrogenation of ethanol and the role of redox centers seems to be important. The activity of Cu-SBA-15 and Cu-MnSBA-15 catalysts significantly increased as a result of oxygen admission to the reaction mixture. The pretreatment of the copper catalyst (Cu-SBA-15) with oxygen led to an increase in Lewis basicity and a decrease in LAS, but reduced the dehydrogenation activity (the reaction without oxygen in the reagents flow). Taking this into account, we propose an ethoxide pathway for the reaction carried out at 473 K in the presence of ethanol and oxygen in the reaction mixture, similar to that postulated as the first step of oxidation of alcohols-to-acids (via aldehyde formation) over gold catalysts [45]. In such a reaction route BrØnsted or Lewis basic sites (present on the surface of all catalysts applied in this study) abstract proton from OH group in ethanol molecules and lead to the formation of ethoxide ([CH_3_CH_2_O]^−^ anions) (Scheme 2). In the following step, electrons from these ions have to be withdrawn by redox species on the catalyst surface (Cu^δ+^ (1 < δ < 2) cations on Cu-SBA-15 abstract electrons and are reduced to Cu^γ+^ (0 < γ < 1)) giving rise to the formation of ethoxy species chemisorbed on LAS. As LAS are located close to Lewis base sites (CuO) the following abstraction of protons (α-hydrogen) from methylene group (CH_2_) in ethoxy species can easily occur and finally, via anion radicals [CH_3_CHO]^−^, acetaldehyde is formed. The role of oxygen is to recover active centers by the oxidation of Cu^γ+^ (0 < γ < 1) to Cu^δ+^ (1 < δ < 2). In this process O_2_^−^ reactive oxygen species are most probably formed and they are active in the total oxidation of ethanol to CO_2_. Thus, oxygen molecules act as electron transmitters increasing the rate of the reaction step in which [CH_3_CH_2_O]^−^ ethoxide is transformed into ethoxy species. It is also worth mentioning that oxygen could favor the alcohol oxidation to acetaldehyde. The lower activity of Cu-MnSBA-15 than Cu-SBA-15 confirms the proposed reaction path. In Cu-MnSBA-15 the synergistic interaction between copper and manganese weakens the electron transfer between copper cations and [CH_3_CH_2_O]^−^ intermediate and/or oxygen molecules. Moreover, the concentration of surface copper species (XPS results) is much lower on Cu-MnSBA-15.

## 3. Materials and Methods

### 3.1. Materials and Chemicals

The chemicals used in this work were: Pluronic P123 (Poly(ethylene glycol)-block-Poly(ethylene glycol)-block-Poly(ethylene glycol)-block) copolymer (Sigma-Aldrich, Saint Louis, MO, USA), tetraethyl orthosilicate (TEOS) (Sigma-Aldrich, Saint Louis, MO, USA, 98%), hydrochloric acid (POCH S.A. Gliwice, Poland, 35–38% HCl), manganese(II) nitrate hydrate (Sigma-Aldrich, Saint Louis, MO, USA, 99.99%), copper(II) nitrate (Sigma-Aldrich, Saint Louis, MO, USA, 99.99%), sodium borohydride (Sigma Aldrich, Saint Louis, MO, USA, >98%), (3-aminopropyl)trimethoxysilane (APTMS, Sigma Aldrich, St. Louis, MO, USA, 97%), 2-propanol (p.a., StanLab, Lublin, Poland), 2,5-hexanedione (Sigma Aldrich, ≥98%) pyridine (C_5_H_5_N, Sigma Aldrich, 99.8%, glucose (Sigma Aldrich, Saint Louis, MO, USA, ≥99.5%), ethanol (StanLab, Lublin, Poland, 96%) and deionized water.

### 3.2. Preparation of Catalysts

#### 3.2.1. Synthesis of SBA-15

The SBA-15 support was prepared via hydrothermal synthesis, according to a published procedure [46]. Pluronic P123 (Poly(ethylene glycol)-block-Poly(ethylene glycol)-block-Poly(ethylene glycol)-block) copolymer was used as a surfactant and TEOS as a source of silicon. The reactant mixture consisted of water, 0.7 M hydrochloric acid (performed with 35–38% HCl, POCH S.A. Gliwice), Pluronic P123 (Sigma-Aldrich) and TEOS (Sigma-Aldrich), at the molar ratios: 1SiO_2_: 0.005Pluronic P123: 1.45HCl: 124H_2_O. After dissolving Pluronic P123 in an HCl solution, the source of silica was added. The mixture was stirred at 308 K for 20 h, moved into a PP bottle, and heated without stirring at 373 K for 24 h. The solid was filtered, washed with deionized water, and dried at room temperature. The template was removed by calcination at 773 K for 8 h in the air in steady state conditions (temperature rate 6 K min^−1^).

#### 3.2.2. Synthesis of MnSBA-15

The MnSBA-15 mesoporous molecular sieve was synthesized according to procedures described in [18] with some modifications.

Pluronic P123 (4 g) was dissolved in 150 g mL of distilled water. Then, the pH was adjusted to 3 by adding HCl (35–38%) (solution A). Tetraethyl orthosilicate (TEOS) (9 g) was mixed with manganese(II) nitrate (Si/Mn = 4) in a separate container (solution B). Then, solution B was added dropwise to solution A and the combined mixture was vigorously stirred at 313 K for 24 h. The resulting mixture was heated at 373 K for 24 h. The obtained solid was precipitated, washed with distilled water, and dried at 373 K. In the last step, the template was removed by calcination at 813 K for 8 h at a heating rate of 1 K min-1. The prepared sample was denoted as MnSBA-15.

#### 3.2.3. Modification of SBA-15 and MnSBA-15 Supports with Copper

The modification of SBA-15 and MnSBA-15 supports with copper was performed according to the two-step procedure proposed by Mou [47]. First, MnSBA-15 was functionalized with APTMS ((3-aminopropyl)-trimethoxysilane) (2.5 mL for 1 g of the support) [14,48]. The obtained functionalized SBA-15 and MnSBA-15 were next stirred for 1 h at room temperature with an aqueous solution of copper nitrate (Cu(NO_3_)_2_) as a metal precursor (in amounts needed to get a final content of 2.2 wt.% of Cu). After filtration and washing, the precipitation was stirred for 20 min with 40 mL of 0.1 M NaBH_4_ solution used as a reducing agent. The solid product was recovered by filtration and washing with water. The final materials were obtained by drying at 373 K and calcination at 773 K for 4 h.

### 3.3. Catalysts Characterization

The actual copper and manganese contents in the catalysts were determined with the use of Inductively Coupled Plasma Optical Emission Spectrometry with an ICP-OES SPECTRO BLUE TI spectrometer (Kleve, Germany). The metals were extracted from the sample by means of HF acid in a microwave oven.

The XRD patterns were recorded on a D8 Advance diffractometer (Bruker) (Billerica, MA, USA) using Cu Kα radiation (λ = 0.154 nm), with a step size of 0.05° in the small-angle range (1°–6°) and with a step size of 0.2° in the wide-angle range (20°–60°).

The nitrogen adsorption-desorption isotherms of all catalysts were measured at 77 K using a Micromeritics ASAP 2020 Physisorption Analyzer (Norcross, GA, USA). Before measurements, the samples were degassed at 573 K for 8 h. The surface area of the materials obtained was calculated with the use of the Brunauer-Emmett-Teller (BET) method. The pore volume and diameter were determined by the DFT method appropriate for mesoporous silicas with a cylindrical pore system using MicroActive software (Version 4.0) from Micromeritics.

Uv–vis spectra were recorded using a Varian-Cary 300 Scan Uv–visible spectrophotometer (Candela, Warszawa, Poland). Powder samples were placed in a cell equipped with a quartz window. The spectra were recorded in the range from 800 to 190 nm. Spectralon was used as the reference material.

X-ray Photoelectron Spectroscopy (XPS) was performed on an ultra-high vacuum photoelectron spectrometer based on a Phoibos150 NAP analyzer (Specs, Berlin, Germany). The analysis chamber was operated under vacuum with a pressure close to 5 × 10^−9^ mbar and the sample was irradiated with a monochromatic Al Kα (1486.6 eV) radiation (15 kV; 10 mA). The spectra were recorded with a flood gun acting as a neutralizer. Binding energies were referenced to the Si 2p peak from silicon to the assumed value of 103.4 eV.

The EPR measurements were recorded using an X-band spectrometer type SE/X 2547 RADIOPAN. The operating microwave frequency was in the range of 8.9 GHz with a magnetic field modulation of 100 kHz. The EPR investigations were performed at 293 K and 77 K. The spectra were recorded for fresh samples and after evacuation in a vacuum at 623 K for 2 h. Additional spectra were taken after the adsorption of NO and used as a probe molecule.

### 3.4. Acidity Measurements

Pyridine (Py) adsorption was measured with a Bruker Invenio S spectrometer (Billerica, MA, USA) at room temperature in the range from 4000 to 400 cm^−1^. The samples pressed at ca. 30 bar into thin wafers with a density of ca. 8–10 mg∙cm^−2^ were placed inside a vacuum cell. Before measurements, the catalysts were evacuated at 623 K for 2 h. Then, Py was introduced into the cell at 423 K and after the saturation, the excess of pyridine was removed by degassing in a vacuum at 423 K for 5 min. Next, the samples were degassed at 423, 473, 523, and 573 K under vacuum for 30 min at each temperature. The spectrum without adsorbed pyridine (after activation) was subtracted from all recorded spectra. The number of Lewis acidic sites were calculated assuming the extinction coefficient ε equal to 2.22 µmol^−1^ cm for the band at 1450 cm^−1^ [34].

The 2-propanol dehydration and dehydrogenation were performed using a microcatalytic pulse reactor inserted between the sample inlet and the column of an SRI 310C gas chromatograph (Torrance, CA, USA). A portion of 0.05 g of the granulated catalyst was activated at 623 K for 2 h under nitrogen flow (40 cm^3^ min^−1^). Additionally, the experiment with pretreatment of Cu-SBA-15 in oxygen (3 cm^3^ min^−1^) and nitrogen (37 cm^3^ min^−1^) flow was performed. The 2-propanol conversion was studied at different temperatures (423–573 K) using 3 μl pulses of alcohol under nitrogen flow (40 cm^3^ min^−1^). The substrate was vaporized before being passed through the catalyst bed with the flow of nitrogen carrier gas. The reaction mixture was separated on a 2-m column filled with Carbowax 400, loaded on a Chromosorb W (80–100 mesh), and the reaction products were detected by FID.

The reaction of cyclization and dehydration of 2,5-hexanedione was performed in a tubular, down-flow reactor. A portion of 0.05 g of each catalyst was placed in the reactor and activated for 2 h at 623 K under nitrogen flow (40 cm^3^ min^−1^). Afterwards, a portion of 0.5 cm^3^ of 2,5-hexanedione was passed continuously over the catalyst at 623 K. The substrate was delivered with a pump system and vaporized before it was passed through the catalyst bed in the presence of a flow of nitrogen carrier gas (40 cm^3^ min^−1^). Reaction products were collected downstream of the reactor in the cold trap (mixture of 2-propanol and liquid nitrogen) and analyzed by gas chromatograph (SRI 310C with a DB-1 column 30 m) equipped with a TCD detector under helium as a carrier gas.

### 3.5. Ethanol Dehydrogenation–FTIR Study in Flow System

The catalysts in the form of a pressed wafer of ca. 6 mg∙cm^−2^ were placed inside the IR reactor cell (HT IRS 01 IR reactor-MeasLine) with CaF_2_ windows. IR spectra were recorded in the range from 4000 to 400 cm^−1^ with a Bruker Vertex 70 FTIR spectrometer. The catalyst surface and gas phase were simultaneously monitored under the reaction conditions. Spectra were corrected by subtracting the spectrum of the activated sample. The spectra presented in this article are the results of this subtraction.

Prior to each experiment, the catalyst was activated in argon flow (40 cm^3^ min^−1^) at 623 K for 2 h, followed by cooling to the reaction temperature 473 or 593 K. Next, after cooling the catalyst to the reaction temperature 473 or 593 K, the mixture of ethanol (12 cm^3^ min^−1^), and argon (28 cm^3^ min^−1^), or ethanol (12 cm^3^ min^−1^), oxygen (3 cm^3^ min^−1^), and argon (25 cm^3^ min^−1^) was passed through the reactor for 120 min. After that, the reagents were cut off and the catalyst was in argon flow (40 cm^3^ min^−1^) at the reaction temperature (desorption process). For Cu-SBA-15, additional experiments were performed. After activation of the sample in Ar flow (40 cm^3^ min^−1^) at 623 K, the catalyst was pretreated in oxygen (3 cm^3^ min^−1^) and argon (37 cm^3^ min^−1^) or ethanol (12 cm^3^ min^−1^) and argon (28 cm^3^ min^−1^) at 473 K for 45 min.

## 4. Conclusions

Thanks to the absence of BAS in all catalysts studied and a relatively weak Lewis acidity, the dehydration of ethanol to ethene (a reaction competitive to dehydrogenation to acetaldehyde) does not occur on copper catalysts applied in this study.

Two different reaction pathways for ethanol dehydrogenation on Cu-SBA-15 are proposed, depending on the temperature and the presence or absence of oxygen in the reagents flow. Without oxygen in the gas flow, the reaction route via dissociative chemisorption of ethanol on LAS-LBS pair and ethoxy species formation occurs, but it works only at the higher reaction temperature (593 K).

In the presence of oxygen, the catalysts became active also at the lower reaction temperature (473 K) and the ethoxide reaction pathway of the process has been proposed. Oxygen plays the role of electron transmitter, enhancing the redox activity in Cu^δ+^/Cu^γ+^ system involved in electron abstraction from [CH_3_CH_2_O]^−^ ions, increasing the rate of ethoxy species formation and adsorption on LAS. This seems to be a step limiting the total reaction rate and therefore, admission of oxygen significantly increased the catalyst activity in the dehydrogenation of ethanol but decreased its selectivity to acetaldehyde by the competitive reaction pathway–total oxidation to CO_2_.

## Figures and Tables

**Table 1:** The chemical composition and textural properties of the catalysts.

Entry	Catalyst	wt.% Cu ^a^	wt.% Mn ^a^	BET Surface Area, m^2^ g^–1^	Total Pore Volume, DFT, cm^3^ g^–1^	Average Pore Diameter, DFT, nm
1.	SBA-15	–	–	868	1.04	10.6
2.	Cu-SBA-15	2.0	–	422	0.57	9.6
3.	MnSBA-15 ^b^	–	0.50	936	1.00	11.2
4.	Cu-MnSBA-15 ^b^	2.2	0.48	661	0.64	10.6

^a^ Copper and manganese loading determined by ICP-OES. ^b^ from [10].

**Table 2:** Parameters of EPR spectra (g factors and hyperfine splittings A [G]).

Entry	Cu-SBA-15	g_ǁ_	A_ǁ_ [G]
1.	fresh sample	2.385	130.5
2.	sample after evacuation at 623 K	2.324	150.7
	and NO ads.		

**Table 3:** The number of Lewis acid sites (LAS) occupied by pyridine after adsorption at 423 K and desorption at different temperatures (calculated on the basis of the extinction coefficient from [34], ε 1450 = 2.22 µmol^−1^ cm).

Entry	Catalyst	Evacuation Temp., K	Number of LAS, μmol g^−1^	Pyridine Desorbed at 573 K from LAS,% ^a^
1.	Cu-SBA-15	523	30.2	
		573	19.2	37
2.	MnSBA-15 ^b^	523	8.8	
		573	4.6	48
3.	Cu-MnSBA-15 ^b^	523	40.6	
		573	32.8	19

^a^ Related to the amount of pyridine chemisorbed after evacuation at 523 K. ^b^ from [10].

**Table 4:** Results of 2-propanol decomposition at 573 K.

Entry	Catalyst	2-Propanol Conv., %	Selectivity, %		
			Propene	Acetone	Ether
1.	SBA-15	1	traces	-	-
2.	Cu-SBA-15	17	66	14	20
3.	Cu-SBA-15-O_2_	18	45	55	0
4.	MnSBA-15 ^a^	8	91	6	3
5.	Cu-MnSBA-15	15	59	40	1

^a^ from [10].

**Table 5:** Results of 2,5-hexanedione cyclization and dehydration at 623 K.

Entry	Catalyst	2,5-Hexanedione Conv., %	Selectivity, %		
			DMF	MCP	MCP/DMF
1.	SBA-15	2	45	55	1.2
2.	Cu-SBA-15	8	72	28	0.4
3.	MnSBA-15	10	5	95	19.0
4.	Cu-MnSBA-15	17	23	77	3.3

## Data Availability

The data presented in this study are available on request from the corresponding author via e-mail: sobiza@amu.edu.pl (I.S.).

## Supplementary Materials

The following supporting information can be downloaded at: https://www.mdpi.com/article/10.3390/ijms24032252/s1.
