# Supplementary material for: Activation of Ethanol Transformation on Copper-Containing SBA-15 and MnSBA-15 Catalysts by the Presence of Oxygen in the Reaction Mixture
Source: Int J Mol Sci. 2023 Jan 23;24(3):2252. doi: 10.3390/ijms24032252 (PMC9916688; doi:10.3390/ijms24032252)
Supplement: Supplementary file 1 [file ijms-24-02252-s001.zip › ijms-2131763-supplementary.pdf]

Supplementary Materials

For Int. J. Mol. Sci.

Activation of Ethanol Transformation on Copper Containing SBA-15 and MnSBA-15 Catalysts by the Presence of Oxygen in the Reaction Mixture

Izabela Sobczak, Joanna Wiśniewska, Piotr Decyk, Maciej Trejda and Maria Ziolek

Faculty of Chemistry, Adam Mickiewicz University in Poznan, Uniwersytetu Poznanskiego 8, 61-614 Poznan, Poland

* Corresponding: sobiza@amu.edu.pl

List of content:

Figure S1. XRD patterns of SBA-15 and MnSBA-15 materials before and after modification with copper. (A, B) The range of 0.6° – 8° 2θ; (C) The range of 30° – 60° 2θ.
Figure S2. Nitrogen adsorption/desorption isotherms of SBA-15 and MnSBA-15 materials before and after modification with copper.
Figure S3. FTIR spectra after (a) adsorption of pyridine at 423 K on MnSBA-15, Cu-MnSBA-15 and Cu-SBA-15 materials and desorption at (b) 423 K, (c) 473 K, (d) 523 K, (e) 573 K for 30 min at each temperature.
Figure S4. FTIR spectra recorded in ethanol flow at 593 K during 120 min for Cu-SBA-15 catalyst (4000-1300 cm^{-1} range).
Figure S5. FTIR spectra of catalysts in the hydroxyl groups region (4000-2500 cm^{-1}) after activation in argon flow at 623 K.
Figure S6. FTIR spectra recorded in argon flow at 473 K during 60 min for Cu-SBA-15 catalyst (4000-1300 cm^{-1} range).
Figure S7. The comparison of FTIR spectra recorded in ethanol and oxygen flow at 473 K for Cu-SBA-15 after activation in Ar or Ar+O_2 flow after 15, 30, 45 and 90 min of the reaction (4000-1300 cm^{-1} range).
Figure S8. The comparison of FTIR spectra recorded in ethanol flow at 473 K for Cu-SBA-15 after activation in Ar or Ar+O_2 flow after 15, 30, 45 and 90 min (4000-1300 cm^{-1} range).

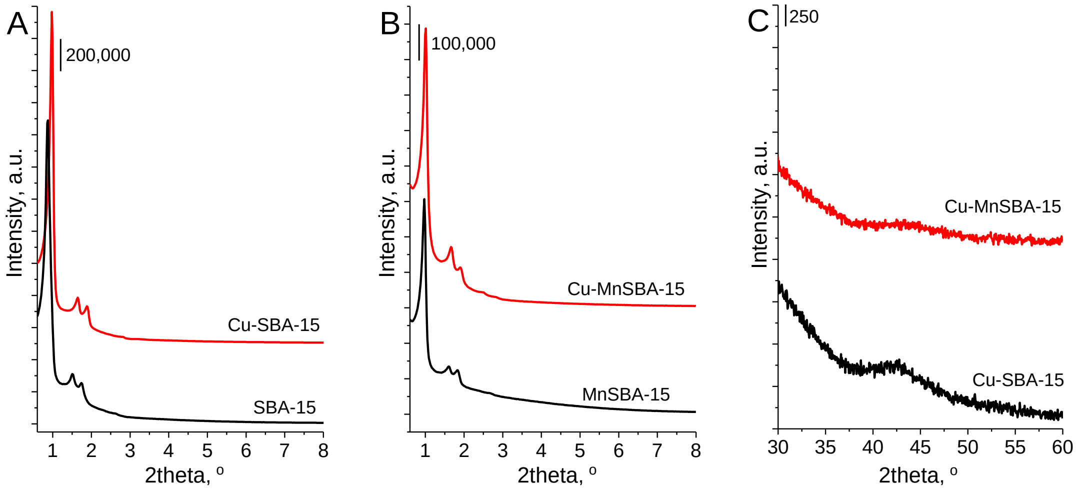

Figure S1. XRD patterns of SBA-15 and MnSBA-15 materials before and after modification with copper. (A, B) The range of 0.6° – 8° 2θ; (C) The range of 30° – 60° 2θ.

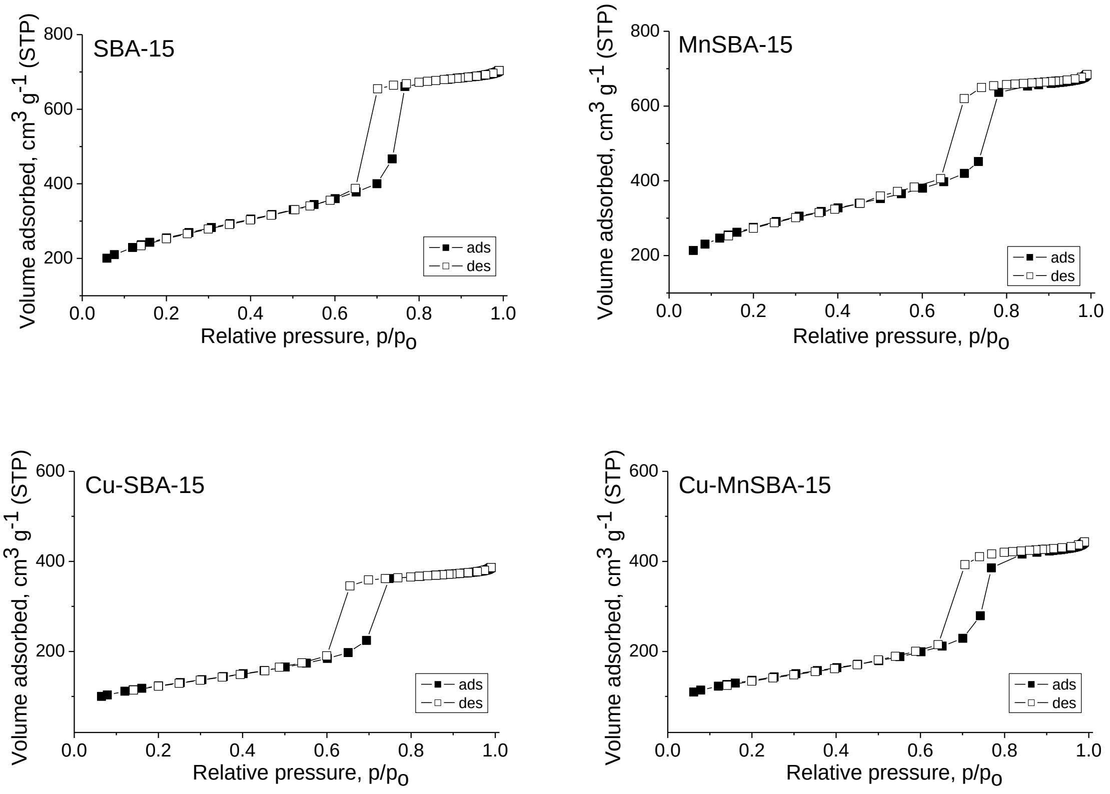

Figure S2. Nitrogen adsorption/desorption isotherms of SBA-15 and MnSBA-15 materials before and after modification with copper.

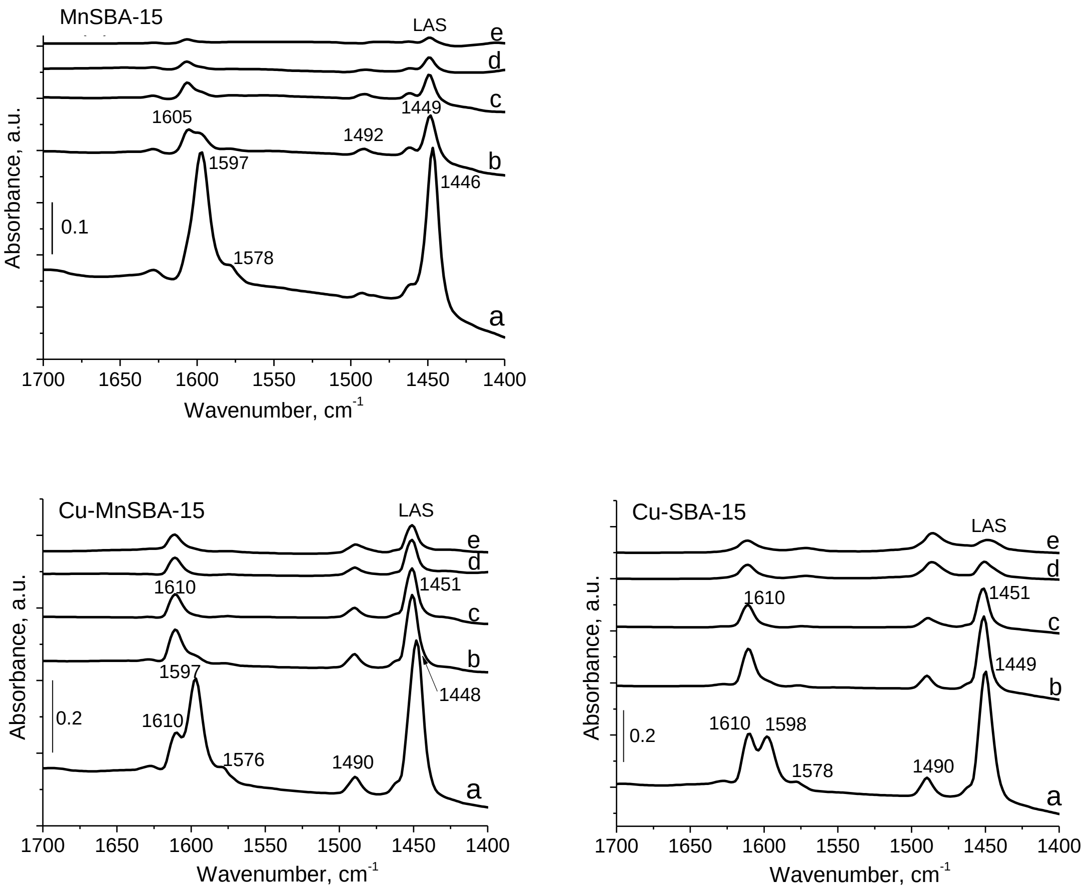

Figure S3. FTIR spectra after (a) adsorption of pyridine at 423 K on MnSBA-15, Cu-MnSBA-15 and Cu-SBA-15 materials and desorption at (b) 423 K, (c) 473 K, (d) 523 K, (e) 573 K for 30 min at each temperature.

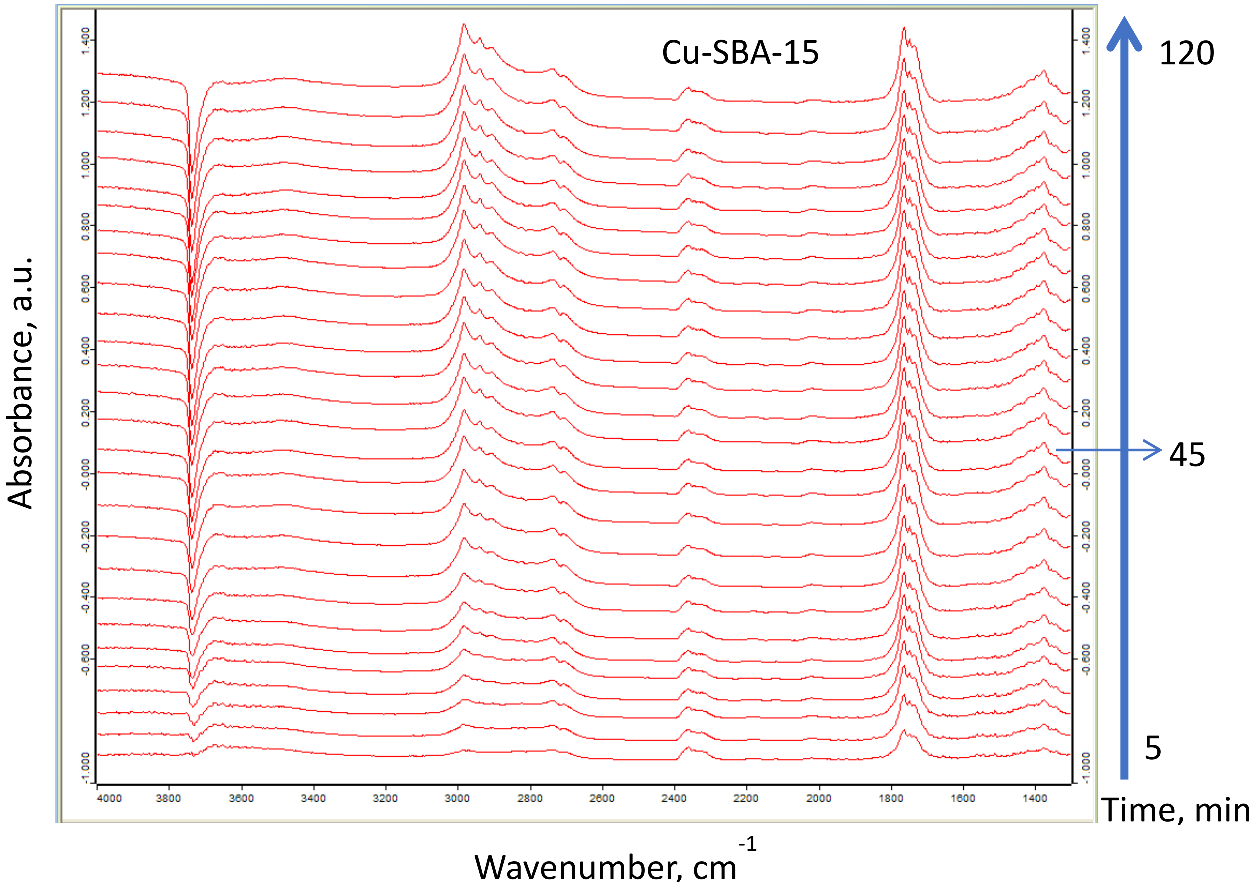

Figure S4. FTIR spectra recorded in ethanol flow at 593 K during 120 min for Cu-SBA-15 catalyst (4000-1300 cm^{-1} range).

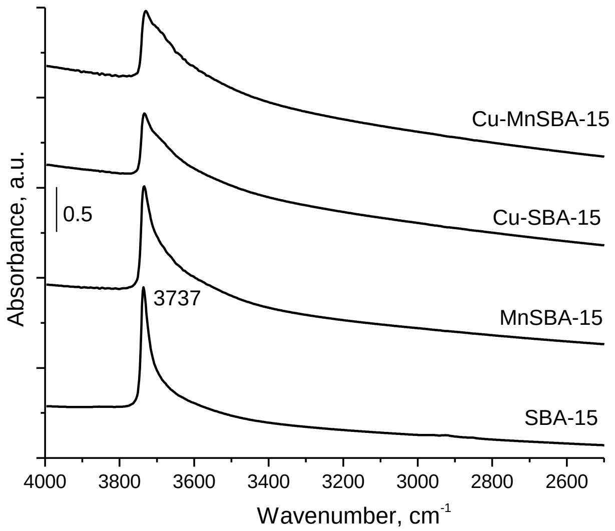

Figure S5. FTIR spectra of catalysts in the hydroxyl groups region (4000-2500 cm^{-1}) after activation in argon flow at 623 K.

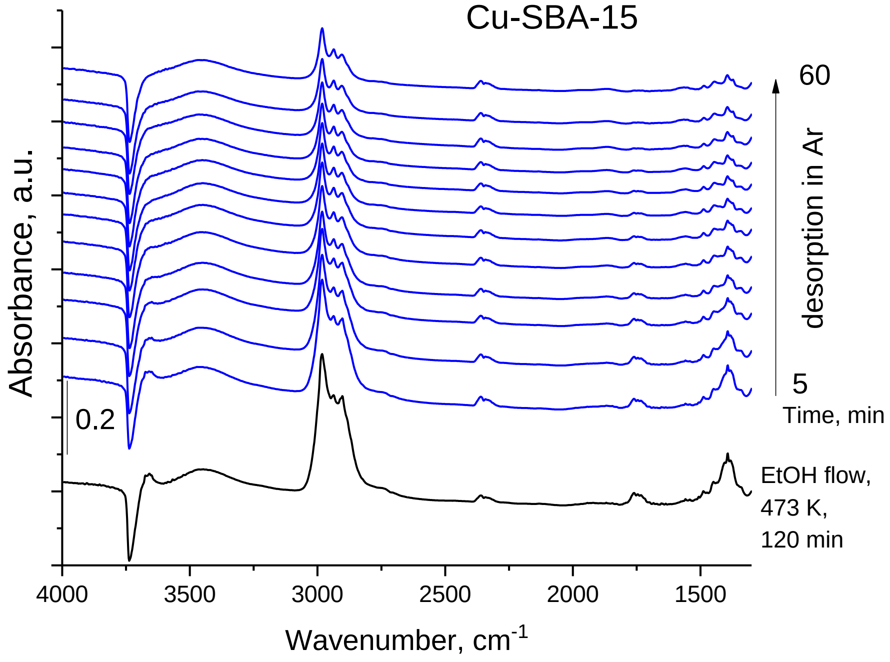

Figure S6. FTIR spectra recorded in argon flow at 473 K during 60 min for Cu-SBA-15 catalyst (4000-1300 cm^{-1} range).

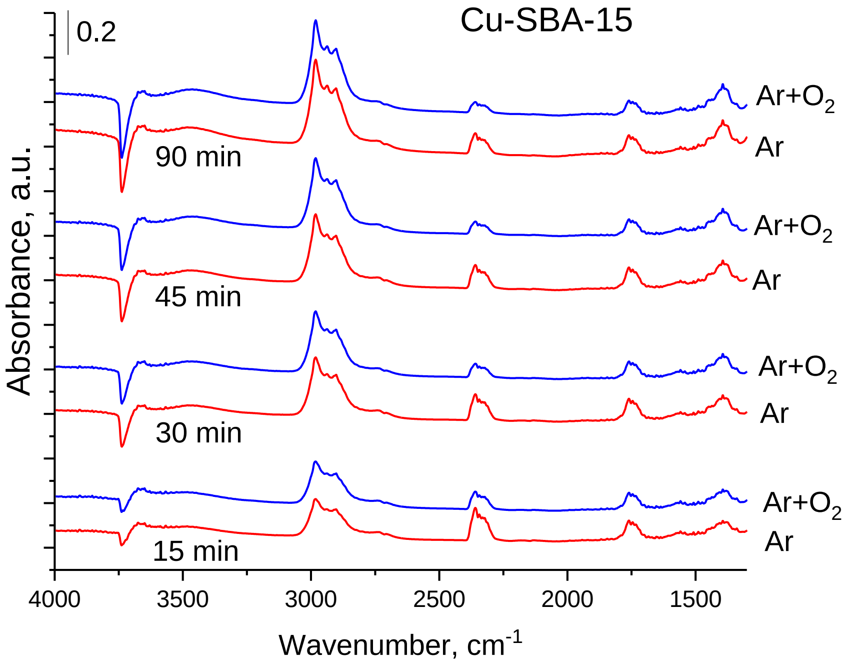

Figure S7. The comparison of FTIR spectra recorded in ethanol and oxygen flow at 473 K for Cu-SBA-15 after activation in Ar or Ar+O_2 flow after 15, 30, 45 and 90 min of the reaction (4000-1300 cm^{-1} range).

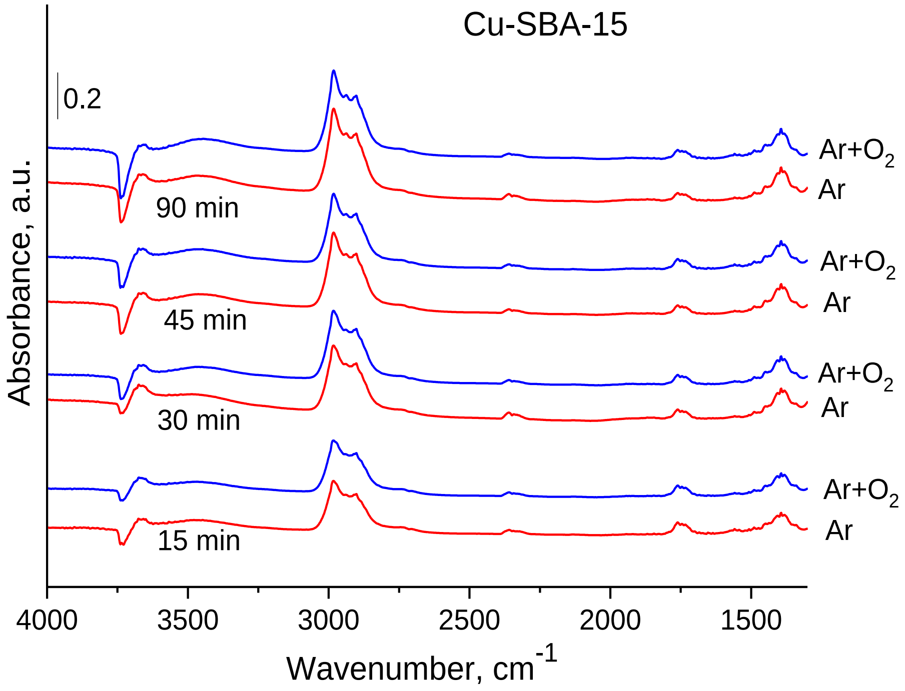

Figure S8. The comparison of FTIR spectra recorded in ethanol flow at 473 K for Cu-SBA-15 after activation in Ar or Ar+O_2 flow after 15, 30, 45 and 90 min (4000-1300 cm^{-1} range).
